# Adaptation of the tetracycline-repressible system for modulating the expression of essential genes in *Cryptococcus neoformans*

**DOI:** 10.1128/msphere.01018-24

**Published:** 2025-05-01

**Authors:** Ci Fu, Nicole Robbins, Leah E. Cowen

**Affiliations:** 1 Department of Molecular Genetics, University of Toronto 204248, Toronto, Ontario, Canada; University of Wisconsin-Madison, Madison, Wisconsin, USA

**Keywords:** tetracycline-repressible system, *Cryptococcus neoformans*, essential gene, Hsp90, Fks1, genetic resource, fungal pathogen

## Abstract

The opportunistic human fungal pathogen *Cryptococcus neoformans* has an enormous impact on human health as the causative agent of cryptococcal meningitis, and there is a dire need to expand our current antifungal arsenal. Essential gene products often serve as ideal targets for antimicrobials, and identifying and characterizing essential genes in a pathogen of interest is critical for drug development. Unfortunately, characterization of essential genes in *C. neoformans* is limited due to its haploid nature and lack of genetic tools for generating effective conditional-expression mutants. To date, the copper-repressible promoter *pCTR4* is the most widely used system to regulate essential gene expression; however, its expression is leaky and copper has pleiotropic effects. In diverse fungal species, including *Saccharomyces cerevisiae*, *Candida albicans*, and *Candida auris*, the tetracycline-repressible promoter system is a powerful tool to regulate gene expression; however, it has yet to be adapted for *C. neoformans*. In this study, we successfully implemented the tetracycline-repressible system in *C. neoformans* to regulate the expression of the essential genes *HSP90* and *FKS1*. Supplementation of cultures with the tetracycline analog doxycycline efficiently depleted *HSP90* at both transcript and protein levels and inhibited *C. neoformans* growth and viability. Similarly, the depletion of *FKS1* with doxycycline enhanced sensitivity of the strain to the echinocandin caspofungin, an antifungal that targets the glucan synthase but is generally ineffective against *C. neoformans*. Thus, this work unveils a novel approach to generate conditional-expression mutants in *C. neoformans,* providing unprecedented potential to systematically study essential gene function in this important human fungal pathogen.

**IMPORTANCE:**

Invasive fungal infections cause millions of deaths annually, while the number of antifungals available to combat these pathogens is limited to only three classes: polyenes, azoles, and echinocandins. The largest source of novel antifungal drug targets are essential gene products, which are required for cellular viability. However, tools to identify and characterize essential genes in *C. neoformans* are extremely limited. Here, we adapted the tetracycline-repressible promoter system, that has been widely used in other organisms, to study essential gene function in *C. neoformans*. By placing this regulatable promoter upstream of the essential genes *HSP90* and *FKS1*, we confirmed that the growth of the strains in the presence of the tetracycline analog doxycycline results in the depletion of essential gene expression. This approach provides a significant advance for the systematic study of essential genes in *C. neoformans*.

## OBSERVATION

Invasive fungal pathogens infect approximately 6.5 million people and directly contribute to 2.5 million deaths annually worldwide (1). Among the most problematic fungal pathogens is *Cryptococcus neoformans,* which causes 147,000 deaths each year (1). Mortality rates remain high, in part, because the number of drug classes that have distinct targets in fungi is limited, and the utility of current antifungal drugs is compromised by either dose-limiting host toxicity or antifungal resistance (2). Fungal-specific essential genes provide attractive drug targets as the majority of antimicrobials in clinical use target functions essential for pathogen viability. While genome-scale mutant collections that include coverage of essential genes are available in the model yeast *Saccharomyces cerevisiae* as well as the fungal pathogen *Candida albicans* (3–5)*,* analogous tools in *C. neoformans* remain limited. *C. neoformans* deletion collections have been employed to further our understanding of its biology (6, 7); however, such resources only cover 62% of predicted protein-coding genes and exclude essential genes (7). Furthermore, tools available for characterizing essential genes in *C. neoformans* are limited. Galactose-inducible and copper-repressible promoters can control *C. neoformans* gene expression (8, 9); however, leaky expression, interference of galactose and copper with *C. neoformans* metabolism and virulence, and difficulty regulating expression in animal hosts limit their utility (10–12).

**Fig 1:**
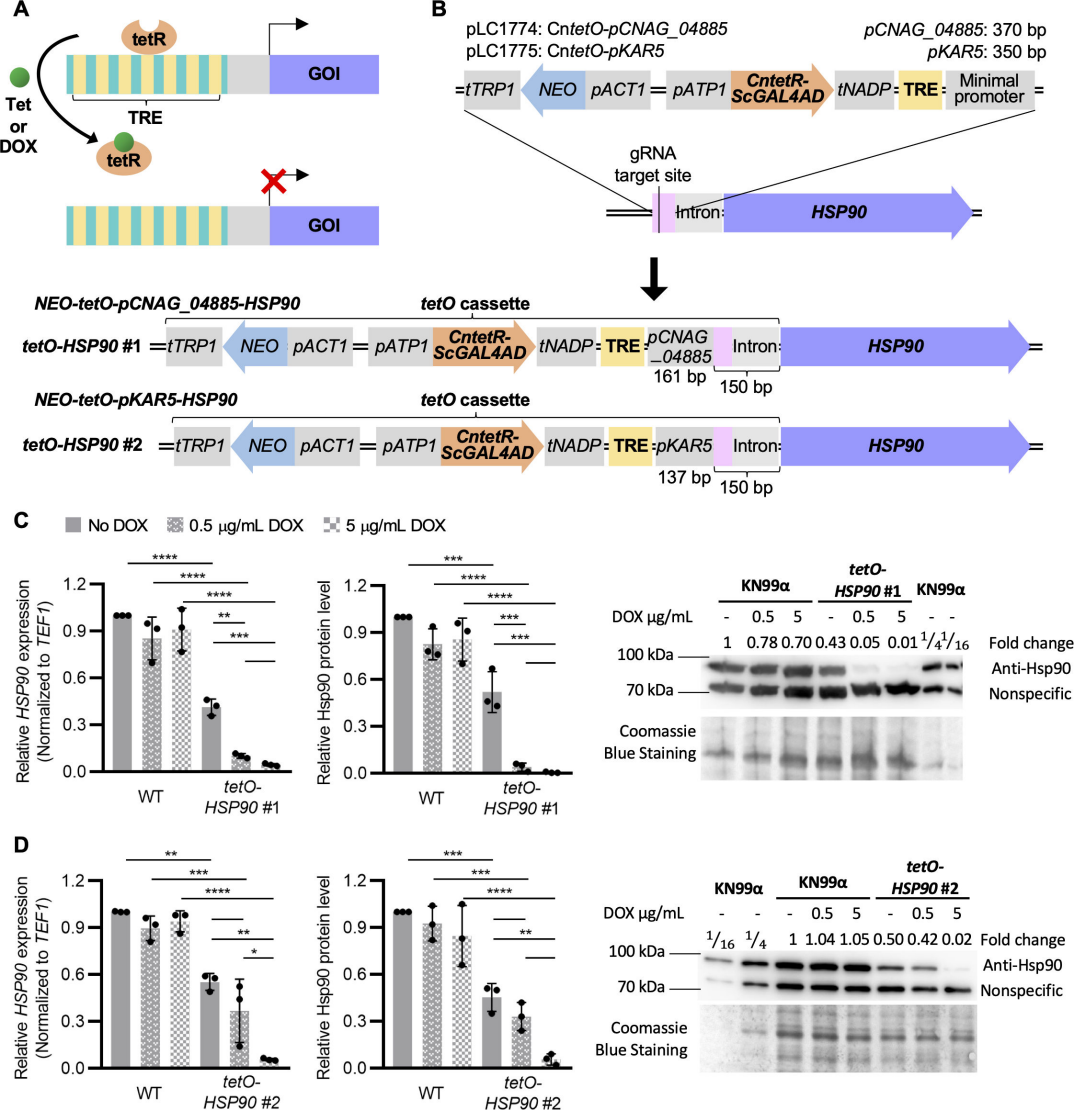
Adaptation of the tetracycline-repressible system in *Cryptococcus neoformans* for regulating gene expression. (**A**) Schematic for the tetracycline-repressible system. In the presence of tetracycline (Tet) or doxycycline (DOX), gene expression is repressed due to disassociation of the transactivator (tetR) from the tetracycline response elements (TRE). (**B**) Schematic of the tetracycline-repressible system in *C. neoformans* for the generation of two *tetO-HSP90* strains. A *C. neoformans* G418 selection marker under the control of the *ACT1* promoter, the *C. neoformans* codon optimized transactivator (tetR) with the activation domain from *S. cerevisiae GAL4*, the tetracycline response element (TRE) containing seven repeats of tetracycline operator sequence, and minimal promoter from either *CNAG_04885* or *KAR5* were assembled into two *tetO* vectors, pLC1774 and pLC1775. *CntetO* cassettes were integrated in the promoter region of *HSP90* via CRISPR/Cas9 genome editing. Doxycycline-sensitive *tetO-HSP90* strains (tetO-*HSP90* #1 and tetO-*HSP90* #2) were identified, and Sanger sequencing confirmed the integration of the tetO cassettes 150 bps upstream of the *HSP90* start codon. The minimal promoters *pCNAG_04885* and *pKAR5* were truncated to 161 bps and 137 bps, respectively. (**C and D**) Wild type (WT, KN99α) and the two *tetO-HSP90* strains were grown overnight in liquid YPD medium in the presence or the absence of 0.5 µg/mL DOX. On the second day, YPD cultures were sub-cultured back into YPD, and YPD with DOX cultures were sub-cultured into YPD containing 0.5 or 5 µg/mL DOX for 4–5 hours at 30°C. Cells were harvested to examine *HSP90* expression levels by quantitative real-time PCR (qRT-PCR) and Hsp90 protein levels by western blot for (**C**) *tetO-HSP90* #1 and (**D**) *tetO-HSP90* #2. Relative expression levels for *HSP90* were normalized to *TEF1* and compared to wild-type cells grown in YPD medium. Each data point represents the mean of technical triplicates, normalized to the *HSP90* expression level in wild-type cells grown in YPD medium. Relative Hsp90 protein levels were normalized to the nonspecific protein band recognized by the anti-Hsp90 antibody and compared to wild type grown in YPD medium. Representative western blot image from one biological experiment for each strain is provided to visualize Hsp90 protein levels. Wild-type (KN99α) protein sample was diluted 4- and 16-fold to visualize the decrease in protein band intensity. Hsp90 protein level fold changes are listed above the western blot image, and Coomassie stain of the membrane confirming equal loading is shown below. Error bars in bar graphs represent standard deviation of the mean for biological triplicates. Two-way ANOVA with Bonferroni’s correction was performed to determine statistical significance. *P ≤ 0.05, **P ≤ 0.01, ***P ≤ 0.001, ****P ≤ 0.0001.

Compared to other regulatable expression systems, the tetracycline-repressible promoter system has superior expression control and compatibility in laboratory settings and infection models. This regulatable promoter system has been successfully adapted in *S. cerevisiae* and other fungal pathogens such as *C. albicans* and *Candida auris* (3, 13, 14). Typically, this system has two components: (i) a chimeric transactivator protein consisting of the *Escherichia coli* tetR DNA-binding domain fused to transcriptional activation domains and (ii) a tetracycline response element (TRE) containing multiple repeats of tetracycline operator sequence to which the transactivator binds (13). Binding of the transactivator protein to the TRE enables constitutive gene expression and the addition of tetracycline (and analogues such as doxycycline (DOX)) blocks association between the transactivator and TRE, resulting in the repression of a tet-promoter regulated gene (Fig. 1A). Importantly, the addition of DOX can reach a steady state above 1 µg/mL in mouse plasma and major organs by feeding animals with DOX-supplemented drinking water or chow, enabling the study of gene essentiality *in vivo* (4, 15, 16). Despite superior *in vitro* and *in vivo* expression regulation, the tetracycline-repressible system has yet to be adapted in *C. neoformans*.

In this observation, we describe the adaptation of the tetracycline-repressible system in *C. neoformans* for modulating essential gene expression, focusing on the well-characterized essential genes *HSP90* (*CNAG_06150*) and *FKS1* (*CNAG_06508*) as a proof of concept (11, 17). To do so, we first codon-optimized the tetracycline-responsive transactivator (tetR) with the Gal4 activation domain from *S. cerevisiae* based on *C. neoformans* codon usage (Data S1) (3, 18). This codon-optimized *CntetR-ScGAL4AD* sequence was then cloned into vectors that included a G418-selectable drug marker, tetracycline response element (TRE), and minimal promoters from either *CNAG_04885* (to generate *tetO-HSP90* #1) or *KAR5* (*CNAG_04850*) (to generate *tetO-HSP90* #2) for efficient transcription initiation in *C. neoformans* (Fig. 1B; Data S1). Minimal promoters were selected based on their low expression profiles from transcriptomic profiling studies to avoid potential competing regulation with the tetracycline-repressible system (19, 20).

The assembled *tetO* cassettes were introduced into wild type KN99α via the short homology-directed CRISPR/Cas9 genome-editing technique to replace 111 bps upstream of the first *HSP90* intron (pink box in Fig. 1B) (18, 21). This location was selected to maintain the integrity of the intergenic intron upstream of *HSP90* start codon that is likely required for proper expression. Transformants were phenotypically screened for DOX-dependent growth phenotypes, and one candidate for each *tetO* cassette was selected for downstream characterization (Fig. 1B). Sanger sequencing of the prioritized transformants showed that only 17 bps containing the Cas9 recognition PAM site in the targeted 111 bp-sequence were replaced by *tetO* cassettes. Additionally, both minimal promoters were truncated from 370 to 161 bps for *pCNAG_04855* and from 350 to 137 bps for *pKAR5*. The non-homologous integration occurring downstream of the Cas9-mediated double-strand break was likely facilitated by the NHEJ pathway (Fig. 1B).

To characterize the activity of the *tetO* cassettes in *C. neoformans,* the two *tetO-HSP90* strains were examined for *HSP90* transcript and protein levels in the absence and presence of DOX (Fig. 1C and D). In the absence of DOX, both strains showed significantly reduced *HSP90* transcript and protein levels, which were about half of the wild-type levels. In the presence of 5 µg/mL (~11.2 µM) DOX, these levels were significantly depleted to below 6% of wild-type levels. At a lower DOX concentration of 0.5 µg/mL (~1.12 µM), *HSP90* mRNA and protein levels were significantly depleted in *tetO-HSP90* #1, but not in *tetO-HSP90* #2, indicating a greater sensitivity to DOX with the truncated minimal promoter *pCNAG_04885* (Fig. 1C and D).

**Fig 2:**
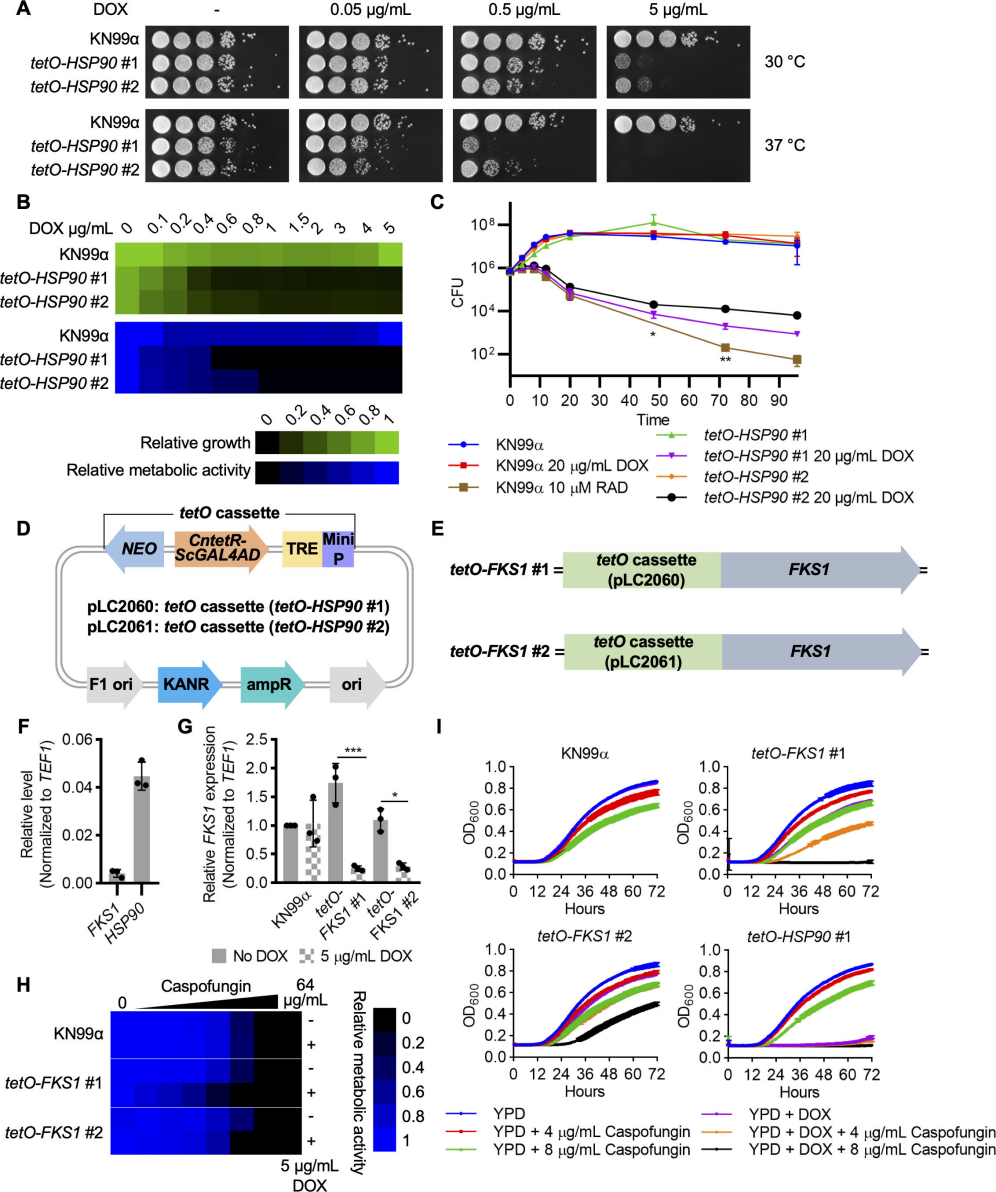
Doxycycline-mediated depletion of *HSP90* and *FKS1* reduces *C. neoformans* viability and enhances caspofungin sensitivity, respectively. (**A**) Cells from wild-type and *tetO-HSP90* overnight liquid YPD cultures were 10-fold serially diluted at a starting optical density (OD_600_) of 0.8 and spotted on YPD agar. Cells from overnight cultures in liquid YPD with 0.5 µg/mL DOX were similarly diluted and spotted on YPD agar supplemented with 0.05, 0.5, or 5 µg/mL DOX. Agar plates were incubated at 30°C or 37°C and imaged after 2 days. (**B**) Cells from overnight wild-type and *tetO-HSP90* cultures in liquid YPD were washed, and cell densities were determined using a hemocytometer. For each strain, 50,000 cells were seeded in 200 µL liquid YPD medium with DOX at a gradient of concentrations ranging from 0 to 5 µg/mL in a 96-well plate, and the plate was incubated at 37°C for 72 hours. OD_600_ was measured using a plate reader, and relative growth was normalized to wild type without DOX. To measure metabolic activity, Alamar blue was added to each well at a dilution factor of 1:40, and the plate was incubated in the dark at room temperature for 12 hours before fluorescence measurement, with an excitation wavelength of 535 nm and an emission wavelength of 595 nm. Relative metabolic activity was normalized to wild type without DOX. (**C**) Wild-type and *tetO-HSP90* strains were inoculated at 1 × 10^6^ CFU in liquid YPD medium with or without 20 µg/mL DOX. Wild type grown in liquid YPD medium supplemented with 10 µM radicicol was used as a control. Cells were grown at 37°C under shaking condition at 220 rpm and plated on YPD agar medium at 0, 4, 8, 12, 20, 48, 72, and 96 hours post inoculation to determine CFUs. Error bars represent standard deviation of the mean for biological triplicates. All three data points (*) and two data points (**) were below the detection limit of 2,000 CFU for wild type grown in the presence of 10 µM radicicol. (**D**) *tetO* cassettes from the *tetO-HSP90* strains were cloned into plasmids pLC2060 and pLC2061. (**E**) *tetO* cassettes were integrated in the promoter region of *FKS1* via CRISPR/Cas9 genome editing to generate *tetO-FKS1* strains (tetO-*FKS1* #1 and tetO-*FKS1* #2). (**F and G**) *HSP90* and *FKS1* expression levels were determined by qRT-PCR for WT (KN99α) (**F**) and *FKS1* expression levels were determined by qRT-PCR for wild type and the two *tetO-FKS1* strains (**G**), as described in Fig. 1C and D. *HSP90* and *FKS1* levels were normalized to *TEF1* in panel F. Error bars in bar graphs represent standard deviation of the mean for biological triplicates. Two-way ANOVA with Bonferroni’s correction was performed to determine statistical significance. *P ≤ 0.05, ***P ≤ 0.001. (**H**) A dose-response assay was performed for KN99α and the two *tetO-FKS1* strains with two-fold dilutions of caspofungin in the absence and presence of 5 µg/mL DOX at 30°C as described in panel B. After 3 days, relative metabolic activity was measured using Alamar blue and normalized to wild type without DOX and caspofungin. (**I**) Growth curve analyses were performed for KN99α, the two *tetO-FKS1* strains, and *tetO-HSP90* #1 with 0, 4, or 8 µg/mL caspofungin in the presence and absence of 5 µg/mL DOX using a CG-12 Cell-Grower Robot, third generation (S&P Robotics) at 30°C. OD_600_ was measured every 15 minutes up to 72 hours with shaking for 1 minute before each measurement. Error bars represent standard deviation of the mean for technical quadruplicates.

To visualize the impact of DOX treatment on *C. neoformans* growth, wild-type and *tetO-HSP90* strains were cultured on both solid YPD agar medium and liquid YPD medium with and without DOX supplementation. Specifically, on YPD agar, 5 µg/mL DOX significantly reduced the growth of both *tetO-HSP90* strains at 30°C and completely blocked growth at 37°C (Fig. 2A). In agreement with the observed DOX regulation of *HSP90* at the transcript and protein levels, 0.5 µg/mL DOX had a more dramatic effect on the growth of *tetO-HSP90* #1 at 37°C compared to *tetO-HSP90* #2 (Fig. 2A). Similar results were observed in liquid medium with levels of DOX below 1 µg/mL reducing growth and metabolic activity of both strains, and *tetO-HSP90* #1 showing increased sensitivity to DOX (Fig. 2B). Finally, we cultured the *tetO-HSP90* strains in the absence and presence of a high concentration of DOX (20 µg/mL) over 4 days and measured viability by quantifying colony-forming units (CFUs) of the surviving populations. Wild-type cells treated with the Hsp90 inhibitor radicicol (RAD) at an inhibitory concentration (10 µM) were used as a control. Similar to RAD treatment, DOX reduced viability of both *tetO-HSP90* strains, with a two-log reduction in CFU compared to wild type and no DOX controls after 72 hours.

To validate the *tetO* system in regulating the expression of an additional essential gene, we cloned the *tetO* cassettes from the two *tetO-HSP90* strains into plasmids pLC2060 and pLC2061 (Fig. 2D and E; Data S1) and integrated the *tetO* cassettes in the promoter region of the essential β-1,3-glucan synthase gene, *FKS1* (22). This gene was selected as it encodes the target of the echinocandin class of antifungals (2) as well as the fact that the gene is expressed approximately 10-fold lower compared with *HSP90* (Fig. 2F). This allowed us to verify the ability of the repressible promoter system to regulate the expression of genes that are endogenously expressed at very different levels as well as to potentially enable the generation of an echinocandin-sensitized strain, given that *C. neoformans* is intrinsically resistant to this antifungal class (2). Addition of 5 µg/mL DOX significantly decreased *FKS1* expression relative to wild-type levels in both *tetO-FKS1* strains (Fig. 2G). In the absence of DOX, *FKS1* was upregulated in *tetO-FKS1* #1 and was expressed at wild-type level in *tetO-FKS1* #2 (Fig. 2G), suggesting expression levels of the target gene in the absence of DOX vary based on endogenous expression of the target gene as well as the minimal promoter downstream of the TRE. Using a dose-response assay and growth curve analysis, we observed that depletion of *FKS1* resulted in hypersensitivity to caspofungin in comparison with the wild type, with enhanced sensitivity noted in *tetO-FKS1* #1 mutant compared with *tetO-FKS1* #2 (Fig. 2H and I).

Compared to our previous use of the copper-repressible *pCTR4* promoter (11), the tetracycline-repressible system presented here represents a powerful tool to study essential gene function in *C. neoformans*. Throughout our optimization, we noted the importance of using a high-fidelity polymerase in amplifying the ~4.5 kb *tetO* cassette to avoid mutations in error-prone sequences in the template, as well as recommend the use of longer homology to facilitate accurate homologous recombination for *tetO* cassette integration (23). We also acknowledge the development of an auxin-inducible degron to degrade essential proteins, including Fks1 in *C. neoformans* with promising results (24). Combination of the adapted *tetO* promoter and the auxin-inducible degron system will likely provide better regulation and enable efficient study of essential genes in *C. neoformans*. Furthermore, in the recent study by Peterson et al., we describe the generation of a marker-free, diploid wild-type *C. neoformans* strain KN99**a**/α, a fusion product of the congenic wild-type strain pair KN99α and KN99**a**, which offers more flexibility in genome editing and enables haploinsufficiency studies in this typically haploid fungal pathogen (25). Collectively, these advances promise a fruitful exploration of essential gene functions in *C. neoformans*.

## SUPPLEMENTAL MATERIAL

Data S1 msphere.01018-24-s0001.pdf Plasmid maps and sequences and Clustal alignment of codon-optimized CntetR-ScGAL4AD sequence in adapted tetO plasmids for generating tetracycline-repressible *C. neoformans* mutants.

ASM does not own the copyrights to Supplemental Material that may be linked to, or accessed through, an article. The authors have granted ASM a non-exclusive, world-wide license to publish the Supplemental Material files. Please contact the corresponding author directly for reuse.
