# Supplementary material for: Adaptation of the tetracycline-repressible system for modulating the expression of essential genes in Cryptococcus neoformans
Source: mSphere. 2025 May 1;10(5):e01018-24. doi: 10.1128/msphere.01018-24 (PMC12108051; doi:10.1128/msphere.01018-24)
Supplement: Data S1 — Plasmid maps and sequences and Clustal alignment of codon-optimized CntetR-ScGAL4AD sequence in adapted tetO plasmids for generating tetracycline-repressible C. neoformans mutants. [file msphere.01018-24-s0001.pdf]

Data S1: Plasmid maps and sequences and Clustal alignment of codon optimized *CntetR-ScGAL4AD* sequence in adapted *tetO* plasmids for generating tetracycline-repressible *Cryptococcus neoformans* mutants.

1. pLC1774

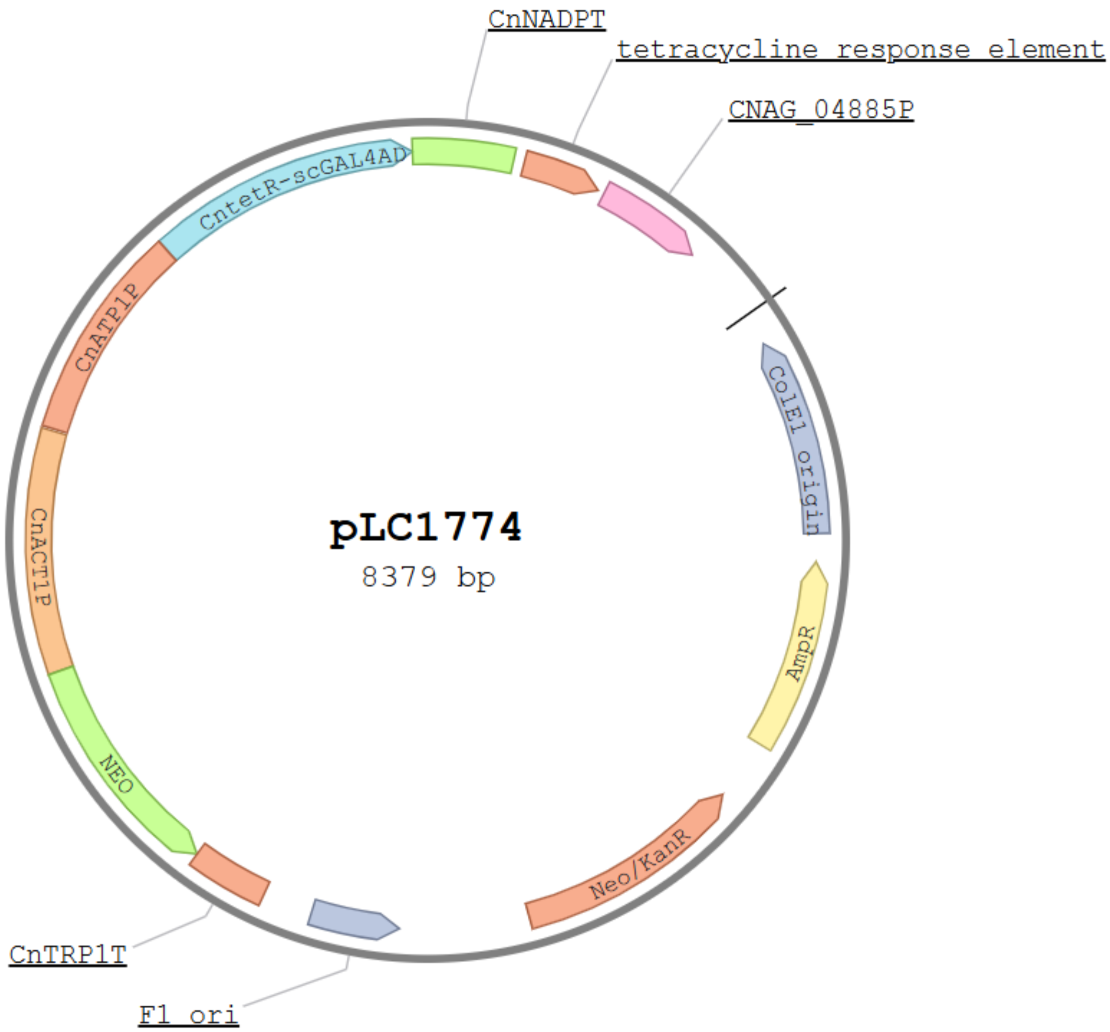

>pLC1774

aaaaaaaaaaaaaagaaaagaaggaacaggagaagaagggaagaagccggtatacagttatttgttcataaagaat
tttatcgcccgtatgcagtttatttgttcatcaagaatttcatCTTTTTATCTGTCACAGTAGCGAGGTCTTCT
TTTTTGAATTTTACCAGTATCGGCTCTTGATCAACCCTTGCCAGCTTCTAGCCACATA
ATCGACAAAACCCATAGCGTCACCCACATTCTGCTCAGTGTTCTCTACCGATTTCAC
CAGTTGCTCATAACGTCCCAGGTTCGTGCGAAATCGATgtttggttcagcaccttgtcgagtttacc
actccctatcagtgatagagaaaagtgaaagtcgagtttaccactccctatcagtgatagagaaaagtgaaagtcgagttt
accactccctatcagtgatagagaaaagtgaaagtcgagtttaccactccctatcagtgatagagaaaagtgaaagtcga

gtttaccactccctatcagtgatagagaaaagtgaaagtcgagtttaccactccctatcagtgatagagaaaagtgaaagt
cgagtttaccactccctatcagtgatagagaaaagtgaaagtcgagcACTAGTAGTCGGTTATGATGACTG
TCCCAGAGAACTATGTCACTCATCATTGGTCTTGAAAGTTTCAGCCGGCCATTCGGT
TCGACGAGGCGGAGAGAAGTCACGCTCATAAGGGAAAACTAGCTACTTCAGCAGC
TTTAGCATTCGATTGACGACAGCAGGAAGTTGCGGCTTGTTGAATTTTAAAAAACGA
GCTAATAGGACCTTTGGTGTGGCAAACCAAGCTGGCAATGTCGTCTTACAATTATCA
AATGCACAGCGAGTGTTGGAACCTGGAGTGCATACATACTTCTATGGAGATTCATCC
TGTAACATGATAGTGGGGCCATATTCCGTTATACACACCAGCTGATATTTTCAAAGAC
GTACCTCGACAATCcatggtcatagctgtttcctgtgtgaaattgttatccgctcacaattccacacaacatacgagc
cggaagcataaagtgtaaagcctggggtgcctaatgagtgagctaactcacattaattgcgttgcgctcactgcccgctttc
cagtcgggaaacctgtcgtgccagctgcattaatgaatcggccaacgcgcggggagaggcggtttgcgtattgggcgctc
ttccgcttcctcgctcactgactcgctgcgctcggtcgttcggctgcggcgagcggtatcagctcactcaaaggcggtaata
cggttatccacagaatcaggggataacgcaggaaagaacatgtgagcaaaaggccagcaaaaggccaggaaccgt
aaaaaggccgcgttgctggcgtttttccataggctccgcccccctgacgagcatcacaaaaatcgacgctcaagtcagag
gtggcgaaacccgacaggactataaagataccaggcgtttccccctggaagctccctcgtgcgctctcctgttccgaccct
gccgcttaccggatacctgtccgcctttctcccttcgggaagcgtggcgctttctcatagctcacgctgtaggtatctcagttcg
gtgtaggtcgttcgctccaagctgggctgtgtgcacgaaccccccgttcagcccgaccgctgcgccttatccggtaactatc
gtcttgagtccaacccggtaagacacgacttatcgccactggcagcagccactggtaacaggattagcagagcgaggta
tgtaggcggtgctacagagttcttgaagtggtggcctaactacggctacactagaagaacagtatttggtatctgcgctctgc
tgaagccagttaccttcggaaaaagagttggtagctcttgatccggcaaacaaaccaccgctggtagcggtggtttttttgttt
gcaagcagcagattacgcgcagaaaaaaaggatctcaagaagatcctttgatcttttctacggggtctgacgctcagtgg
aacgaaaactcacgttaagggattttggtcatgagattatcaaaaaggatcttcacctagatccttttaaattaaaaatgaag
ttttaaatcaatctaaagtatatatgagtaaacttggtctgacagttaccaatgcttaatcagtgaggcacctatctcGgcgat
ctgtctatttcgttcatccatagttgcctgactccccgtcgtgtagataactacgatacgggagggcttaccatctggccccagt
gctgcaatgataccgcgagacccacgctcaccggctccagatttatcagcaataaaccagccagccggaagggccga
gcgcagaagtggtcctgcaactttatccgcctccatccagtctattaattgttgccgggaagctagagtaagtagttcgccag
ttaatagtttgcgcaacgttgttgccattgctacaggcatcgtggtgtcacgctcgtcgtttggtatggcttcattcagctccggtt
cccaacgatcaaggcgagttacatgatcccccatgttgtgcaaaaaagcggttagctccttcggtcctccgatcgttgtcag
aagtaagttggccgcagtgttatcactcatggttatggcagcactgcataattctcttactgtcatgccatccgtaagatgctttt
ctgtgactggtgagtactcaaccaagtcattctgagaatagtgtatgcggcgaccgagttgctcttgcccggcgtcaatacg
ggataataccgcgccacatagcagaactttaaaagtgctcatcattggaaaacgttcttcggggcgaaaactctcaagga
tcttaccgctgttgagatccagttcgatgtaacccactcgtgcacccaactgatcttcagcatcttttactttcaccagcgtttctg
ggtgagcaaaaacaggaaggcaaaatgccgcaaaaaagggaataagggcgacacggaaatgttgaatactcatact
cttcctttttcaattcagaagaactcgtcaagaaggcgatagaaggcgatgcgctgcgaatcgggagcggcgataccgta
aagcacgaggaagcggtcagcccattcgccgccaagctcttcagcaatatcacgggtagccaacgctatgtcctgatag
cggtccgccacacccagccggccacagtcgatgaatccagaaaagcggccattttccaccatgatattcggcaagcag
gcatcgccatgggtcacgacgagatcctcgccgtcgggcatgcgcgccttgagcctgAcgaacagttcggctggcgcga
gcccctgatgctcttcgtccagatcatcctgatcgacaagaccggcttccatccgagtacgtgctcgctcgatgcgatgtttc
gcttggtggtcgaatgggcaggtagccggatcaagcgtatgcagccgccgcattgcatcagccatgatggatactttctcg
gcaggagcaaggtgggatgacaggagatcctgccccggcacttcgcccaatagcagccagtcccttcccgcttcagtga

caacgtcgagcacagctgcgcaaggaacgcccgtcgtggccagccacgatagccgcgctgcctcgtcctgcagttcatt
cagggcaccggacaggtcggtcttgacaaaaagaaccgggcgcccctgcgctgacagccggaacacggcggcatca
gagcagccgattgtctgttgtgcccagtcatagccgaatagcctctccacccaagcggccggagaacctgcgtgcaatcc
atcttgttcaatcatgcgaaacgatcctcatcctgtctcttgatcagatcttgatcccctgcgccatcagatccttggcggcaag
aaagccatccagtttactttgcagggcttcccaaccttaccagagggcgccccagctggcaattccggttcgcttgctgtcc
ataaaaccgcccagtctagctatcgccatgtaagcccactgcaagctacctgctttctctttgcgcttgcgttttcccttgtccag
atagcccagtagctgacattcatccggggtcagcaccgtttctgcggactggctttctacgtgttccgcttcctttagcagccct
tgcgccctgaatttgttaaaattcgcgttaaatttttgttaaatcagctcattttttaaccaataggccgaaatcggcaaaatcc
cttataaatcaaaagaatagaccgagatagggttgagtgttgttccagtttggaacaagagtccactattaaagaacgtgg
actccaacgtcaaagggcgaaaaaccgtctatcagggcgatggcccactacgtgaaccatcaccctaatcaagttttttg
gggtcgaggtgccgtaaagcactaaatcggaaccctaaagggagcccccgatttagagcttgacggggaaagccggc
gaacgtggcgagaaaggaagggaagaaagcgaaaggagcgggcgctagggcgctggcaagtgtagcggtcacgc
tgcgcgtaaccaccacacccgccgcgcttaatgcgccgctacagggcgcgtccattcgccattcaggctgcgcaactgtt
gggaagggcgatcggtgcgggcctcttcgctattacgccagctggcgaaagggggatgtgctgcaaggcgattaagttg
ggtaacgccagggttttcccagtcacgacgttgtaaaacgacggccagtGGTTTATCTGTATTAACACGGAA
GAGATGTAGAAACTAGCTTCCTGGTTTCAGAGACAAGGAGCCATGAAGATCCTGAG
GATTCAACGGCGTTGCTGAGGGTGGGAAGGATGGTATTGTTGTCTGTAGATAGATTA
ATGGGCATATAATGTCAAAAAAGATGATTGACACGATGTCTGTCTCGAATTCGCAGT
GTGCTAAAATTGTATCACTTCTCTAATATGTATATATACACCCTCTAAGGAAAATTAACC
CCTTACCGCCTTCACTCAGAAGAACTCGTCAAGAAGGCGATAGAAGGCGATGCGCT
GCGAATCGGGAGCGGCGATACCGTAAAGCACGAGGAAGCGGTCAGCCCATTCGCC
GCCAAGCTCTTCAGCAATATCACGGGTAGCCAACGCTATGTCCTGATAGCGGTCCG
CCACACCCAGCCGGCCACAGTCGATGAATCCAGAAAAGCGGCCATTTTCCACCATG
ATATTCGGCAAGCAGGCATCGCCATGGGTCACGACGAGATCCTCGCCGTCGGGCA
TGCGCGCCTTGAGCCTGGCGAACAGTTCGGCTGGCGCGAGCCCCTGATGCTCTTC
GTCCAGATCATCCTGATCGACAAGACCGGCTTCCATCCGAGTACGTGCTCGCTCGA
TGCGATGTTTCGCTTGGTGGTCGAATGGGCAGGTAGCCGGATCAAGCGTATGCAG
CCGCCGCATTGCATCAGCCATGATGGATACTTTCTCGGCAGGAGCAAGGTGGGATG
ACAGGAGATCCTGCCCCGGCACTTCGCCCAATAGCAGCCAGTCCCTTCCCGCTTC
AGTGACAACGTCGAGCACAGCTGCGCAAGGAACGCCCGTCGTGGCCAGCCACGA
TAGCCGCGCTGCCTCGTCCTGCAGTTCATTCAGGGCACCGGACAGGTCGATCTTG
ACAAAAAGAACCGGGCGCCCCTGCGCTGACAGCCGGAACACGGCGGCATCAGAG
CAGCCGATTGTCTGTTGTGCCCAGTCATAGCCGAATAGCCTCTCCACCCAAGCGGC
CGGAGAACCTGCGTGCAATCCATCTTGTTCAATCATAGACATGTTGGGCGAGTTTAC
TAATGGAAAAAGACAATTAGCAAAGAGGCTGCATATTAGGAGACGCGGTGAAGATTG
GGGAGGACAATAGGGTCCGTGCACCAAAAAGTCCTGACGGCAGCTCTGTTCAACG
CACATCCATCTCTTCCAGCTATGCGTGTGGCACCAGCACGGATGGATTGTTGCACC
AAAAGGACAAGGCGAGATGGATGCAGGTCGAGGCGATGCGGTGCTATCGGCCCTC
GTTGTCGAACGCCACGTCGTCCTCTCGGCGACCGTCAATCCTGGAAGGAAATGAC
CAAGCGTGCACCCAGCATCCAGCATCCAACATCCAGCACCCAGCACCTAGCACCC

ATGATCCAATGGCCGGCATGCAGATCAGCGTGGGGAGAGCACCATCTAGCAGCCC
CTGGCAGGCCCAGCATTGTGCTCGGATGTGCGATAGGCGGTGGGTGCGATAAGGT
GGATTAGTGGAGGATGGAGGACTGAGTGGCGGGCAAGGGAAGGGGGCGGGAAGA
TGACTCACCTTGGGCAGTGGGTGGGGGATGCGGTATAGGGGATGTATGTGGATGC
TGGCGGAGGATAGAAGCTGTAAGCTGAGCTGGTGCCAACGGTGACGCTGTGAGAG
TGGTCTCGGCGACCCTGACGGCGACTCGGCGTTACCCCGTGTGGCACACCGGAC
GCGCGTCGAGTGACGTTGCTGCTATTGTTGTTACCATCATCCTCTCCTCCGACAAC
CATACACTCAGTGCACACCATGCCACTCGAATCCTGCATGCTTATGTGAGTCCTCCC
CTTCCCGTGCGCCGCTCTCCAGCTCACATCCTCGCAGCGCGGCCGCTTAAAAACG
GGTGGCTGCGCTGCTGCTGGATGTATGGGAAAGATGGAGAGATTGTAGGCTCGGC
GGACTGATTTTCGAAACAGCTAAGAAACGAGAACCACTACCGACAGTCCGGTTTTT
CTTCCAGAAGCGGACATGACCGCTGTTCGCGCGACGATGCCACTCCTTCATTCAGC
AAGAATGAAGCAAGGAAGAACGATGTCATTCCGCACTCTTGGGAAGGAATCCACCA
TCAACAAAAGGTATGGAATAAAACAGATGCTGTGTTGCTAGGAGCTACCTTTTGTTT
CTCATAAAAGATTGTCTATACATATATGGATAATACTAGGCATTGTCCGCTTCAGAGCC
AAGAGAACAAACGGCTGTTTCTCTACATGCCATGCATACTTGTATATATGGATAGCGC
CCCTGCACCGCACCACGATCCTCATCATTACATATCTTCTCTTCCAAGTATGCTGCAT
ATATAGCTCTCGATGCGGTACGCATCGTTGATTGAAACGCTTGGTGGAGTGCTTGAT
GGCCCGAAATGCCACGTGTACAACGCCACAAGCCCGAAAGGTAGGGCCTCGTGTA
AAACGCCGCGATTCGCCGGCCCTCCGTGGTCCCTGTTCGTTCCGTCTctctccctggcgc
cctcgtcctcccccactcccaacatctctttcttccttcatcttccaactccctcaaatccatactcccctctctcaccactcagcc
aaaccaaaaatgtcccgactcgacaagtccaaggtcatcaactccgccctcgagctcctcaacgaggtcggtatcgagg
gtctcaccacccgaaagctcgcccagaagctcggtgtcgagcagcccaccctctactggcacgtcaagaacaagcgag
ccctcctcgacgccctcgccatcgagatgctcgaccgacaccacacccacttctgtcccctcgagggtgagtcctggcag
gacttcctccgaaacaacgccaagtccttccgatgtgccctcctctcccaccgagacggtgccaaggtccacctcggtacc
cgacccaccgagaagcagtacgagaccctcgagaaccagctcgccttcctctgtcagcagggtttctccctcgagaacg
ccctctacgccctctccgccgtcggtcacttcaccctcggttgtgtcctcgaggaccaggagcaccaggtcgccaaggag
gagcgagagacccccaccaccgactccatgccccccctcctccgacaggccatcgagctcttcgaccaccagggtgcc
gagcccgccttcctcttcggtctcgagctcatcatctgtggtctcgagaagcagctcaagtgtgagtccggttcctccaccctc
ttcggtggtgccaacttcaaccagtccggtaacatcgccgactcctccctctccttcaccttcaccaactcctccaacggtcc
caacctcatcaccacccagaccaactcccaggccctctcccagcccatcgcctcctccaacgtccacgacaacttcatga
acaacaccgaccagaccgcctacaacgccttcggtatcaccaccggtatgttcaacaccaccaccatggacgacgtcta
caactacctcttcgacgacgaggacacccccccaaccccaagaaggagtaagctagagataatcaagttgataaatg
gttgtgattagctgtaacaatcattg

2. pLC1775

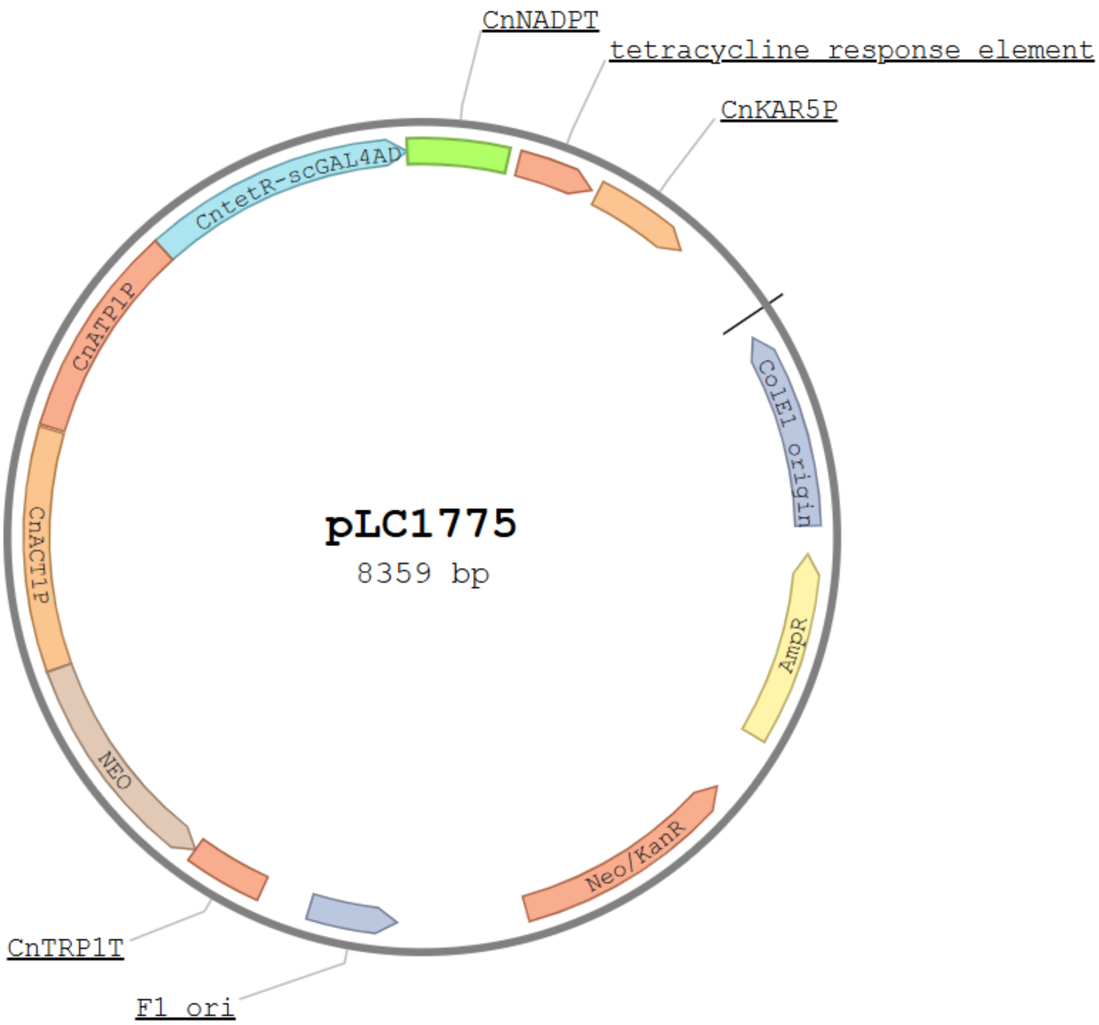

>pLC1775

aaaaaaaaaaaaaagaaaagaaggaacaggagaagaagggaagaagccggtatacagttatttgttcataaagaat
tttatcgcccgtatgcagtttatttgttcatcaagaatttcatCTTTTTATCTGTCACAGTAGCGAGGTCTTCT
TTTTTGAATTTTACCAGTATCGGCTCTTGATCAACCCTTGCCAGCTTCTAGCCACATA
ATCGACAAAACCCATAGCGTCACCCACATTCTGCTCAGTGTTCTCTACCGATTTCAC
CAGTTGCTCATAACGTCCCAGGTTCGTGCGAAATCGATgtttggttcagcaccttgtcgagtttacc
actccctatcagtgatagagaaaagtgaaagtcgagtttaccactccctatcagtgatagagaaaagtgaaagtcgagttt
accactccctatcagtgatagagaaaagtgaaagtcgagtttaccactccctatcagtgatagagaaaagtgaaagtcga
gtttaccactccctatcagtgatagagaaaagtgaaagtcgagtttaccactccctatcagtgatagagaaaagtgaaagt
cgagtttaccactccctatcagtgatagagaaaagtgaaagtcgagcACTAGTTGGAAGATGGAGATATG
GTACAAGAATGGACAGATGGAAACAAGAAATGGGCATCGCTCCTTTAGGGTGGATA

CGTGAGTTTTTGTCAGAGATGAGTGGAAACCCAAAATGACTGTCATCGTAGTCCCG
ATCGGTAGCCCACAAGGAGATGGTCTCCATTTCAAGTCCAATCCAAAGTTTGGGCA
GCATGGAGAGTGGCTGAAAAAGAAAGATTGGCCTACAGAATTGCTTACTGTATAGTT
GAGCAAATATTGTCAGGGGACCAACTGGTTTGTATTATTATCACATGTATTTTTATGAC
GCAATTCCTTGTCGTTTTCCTTTCCACGTTTAGTACTTACCTCTAACGGATCcatggtcat
agctgtttcctgtgtgaaattgttatccgctcacaattccacacaacatacgagccggaagcataaagtgtaaagcctgggg
tgcctaatgagtgagctaactcacattaattgcgttgcgctcactgcccgctttccagtcgggaaacctgtcgtgccagctgc
attaatgaatcggccaacgcgcggggagaggcggtttgcgtattgggcgctcttccgcttcctcgctcactgactcgctgcg
ctcggtcgttcggctgcggcgagcggtatcagctcactcaaaggcggtaatacggttatccacagaatcaggggataacg
caggaaagaacatgtgagcaaaaggccagcaaaaggccaggaaccgtaaaaaggccgcgttgctggcgtttttccat
aggctccgcccccctgacgagcatcacaaaaatcgacgctcaagtcagaggtggcgaaacccgacaggactataaag
ataccaggcgtttccccctggaagctccctcgtgcgctctcctgttccgaccctgccgcttaccggatacctgtccgcctttctc
ccttcgggaagcgtggcgctttctcatagctcacgctgtaggtatctcagttcggtgtaggtcgttcgctccaagctgggctgt
gtgcacgaaccccccgttcagcccgaccgctgcgccttatccggtaactatcgtcttgagtccaacccggtaagacacga
cttatcgccactggcagcagccactggtaacaggattagcagagcgaggtatgtaggcggtgctacagagttcttgaagt
ggtggcctaactacggctacactagaagaacagtatttggtatctgcgctctgctgaagccagttaccttcggaaaaagag
ttggtagctcttgatccggcaaacaaaccaccgctggtagcggtggtttttttgtttgcaagcagcagattacgcgcagaaa
aaaaggatctcaagaagatcctttgatcttttctacggggtctgacgctcagtggaacgaaaactcacgttaagggattttgg
tcatgagattatcaaaaaggatcttcacctagatccttttaaattaaaaatgaagttttaaatcaatctaaagtatatatgagta
aacttggtctgacagttaccaatgcttaatcagtgaggcacctatctcagcgatctgtctatttcgttcatccatagttgcctgac
tccccgtcgtgtagataactacgatacgggagggcttaccatctggccccagtgctgcaatgataccgcgagacccacgc
tcaccggctccagatttatcagcaataaaccagccagccggaagggccgagcgcagaagtggtcctgcaactttatccg
cctccatccagtctattaattgttgccgggaagctagagtaagtagttcgccagttaatagtttgcgcaacgttgttgccattgct
acaggcatcgtggtgtcacgctcgtcgtttggtatggcttcattcagctccggttcccaacgatcaaggcgagttacatgatc
ccccatgttgtgcaaaaaagcggttagctccttcggtcctccgatcgttgtcagaagtaagttggccgcagtgttatcactcat
ggttatggcagcactgcataattctcttactgtcatgccatccgtaagatgcttttctgtgactggtgagtactcaaccaagtcat
tctgagaatagtgtatgcggcgaccgagttgctcttgcccggcgtcaatacgggataataccgcgccacatagcagaactt
taaaagtgctcatcattggaaaacgttcttcggggcgaaaactctcaaggatcttaccgctgttgagatccagttcgatgtaa
cccactcgtgcacccaactgatcttcagcatcttttactttcaccagcgtttctgggtgagcaaaaacaggaaggcaaaatg
ccgcaaaaaagggaataagggcgacacggaaatgttgaatactcatactcttcctttttcaattcagaagaactcgtcaag
aaggcgatagaaggcgatgcgctgcgaatcgggagcggcgataccgtaaagcacgaggaagcggtcagcccattcg
ccgccaagctcttcagcaatatcacgggtagccaacgctatgtcctgatagcggtccgccacacccagccggccacagt
cgatgaatccagaaaagcggccattttccaccatgatattcggcaagcaggcatcgccatgggtcacgacgagatcctc
gccgtcgggcatgcgcgccttgagcctggcgaacagttcggctggcgcgagcccctgatgctcttcgtccagatcatcctg
atcgacaagaccggcttccatccgagtacgtgctcgctcgatgcgatgtttcgcttggtggtcgaatgggcaggtagccgg
atcaagcgtatgcagccgccgcattgcatcagccatgatggatactttctcggcaggagcaaggtgggatgacaggaga
tcctgccccggcacttcgcccaatagcagccagtcccttcccgcttcagtgacaacgtcgagcacagctgcgcaaggaa
cgcccgtcgtggccagccacgatagccgcgctgcctcgtcctgcagttcattcagggcaccggacaggtcggtcttgaca
aaaagaaccgggcgcccctgcgctgacagccggaacacggcggcatcagagcagccgattgtctgttgtgcccagtca
tagccgaatagcctctccacccaagcggccggagaacctgcgtgcaatccatcttgttcaatcatgcgaaacgatcctcat

cctgtctcttgatcagatcttgatcccctgcgccatcagatccttggcggcaagaaagccatccagtttactttgcagggcttc
ccaaccttaccagagggcgccccagctggcaattccggttcgcttgctgtccataaaaccgcccagtctagctatcgccat
gtaagcccactgcaagctacctgctttctctttgcgcttgcgttttcccttgtccagatagcccagtagctgacattcatccggg
gtcagcaccgtttctgcggactggctttctacgtgttccgcttcctttagcagcccttgcgccctgaattttgttaaaattcgcgtta
aatttttgttaaatcagctcattttttaaccaataggccgaaatcggcaaaatcccttataaatcaaaagaatagaccgagat
agggttgagtgttgttccagtttggaacaagagtccactattaaagaacgtggactccaacgtcaaagggcgaaaaaccg
tctatcagggcgatggcccactacgtgaaccatcaccctaatcaagttttttggggtcgaggtgccgtaaagcactaaatcg
gaaccctaaagggagcccccgatttagagcttgacggggaaagccggcgaacgtggcgagaaaggaagggaagaa
agcgaaaggagcgggcgctagggcgctggcaagtgtagcggtcacgctgcgcgtaaccaccacacccgccgcgctta
atgcgccgctacagggcgcgtccattcgccattcaggctgcgcaactgttgggaagggcgatcggtgcgggcctcttcgct
attacgccagctggcgaaagggggatgtgctgcaaggcgattaagttgggtaacgccagggttttcccagtcacgacgtt
gtaaaacgacggccagtGGTTTATCTGTATTAACACGGAAGAGATGTAGAAACTAGCTTCCT
GGTTTCAGAGACAAGGAGCCATGAAGATCCTGAGGATTCAACGGCGTTGCTGAGG
GTGGGAAGGATGGTATTGTTGTCTGTAGATAGATTAATGGGCATATAATGTCAAAAAA
GATGATTGACACGATGTCTGTCTCGAATTCGCAGTGTGCTAAAATTGTATCACTTCTC
TAATATGTATATATACACCCTCTAAGGAAAATTAACCCCTTACCGCCTTCACTCAGAAG
AACTCGTCAAGAAGGCGATAGAAGGCGATGCGCTGCGAATCGGGAGCGGCGATAC
CGTAAAGCACGAGGAAGCGGTCAGCCCATTCGCCGCCAAGCTCTTCAGCAATATCA
CGGGTAGCCAACGCTATGTCCTGATAGCGGTCCGCCACACCCAGCCGGCCACAGT
CGATGAATCCAGAAAAGCGGCCATTTTCCACCATGATATTCGGCAAGCAGGCATCG
CCATGGGTCACGACGAGATCCTCGCCGTCGGGCATGCGCGCCTTGAGCCTGGCG
AACAGTTCGGCTGGCGCGAGCCCCTGATGCTCTTCGTCCAGATCATCCTGATCGAC
AAGACCGGCTTCCATCCGAGTACGTGCTCGCTCGATGCGATGTTTCGCTTGGTGGT
CGAATGGGCAGGTAGCCGGATCAAGCGTATGCAGCCGCCGCATTGCATCAGCCAT
GATGGATACTTTCTCGGCAGGAGCAAGGTGGGATGACAGGAGATCCTGCCCCGGC
ACTTCGCCCAATAGCAGCCAGTCCCTTCCCGCTTCAGTGACAACGTCGAGCACAG
CTGCGCAAGGAACGCCCGTCGTGGCCAGCCACGATAGCCGCGCTGCCTCGTCCT
GCAGTTCATTCAGGGCACCGGACAGGTCGATCTTGACAAAAAGAACCGGGCGCCC
CTGCGCTGACAGCCGGAACACGGCGGCATCAGAGCAGCCGATTGTCTGTTGTGCC
CAGTCATAGCCGAATAGCCTCTCCACCCAAGCGGCCGGAGAACCTGCGTGCAATC
CATCTTGTTCAATCATAGACATGTTGGGCGAGTTTACTAATGGAAAAAGACAATTAGC
AAAGAGGCTGCATATTAGGAGACGCGGTGAAGATTGGGGAGGACAATAGGGTCCG
TGCACCAAAAAGTCCTGACGGCAGCTCTGTTCAACGCACATCCATCTCTTCCAGCT
ATGCGTGTGGCACCAGCACGGATGGATTGTTGCACCAAAAGGACAAGGCGAGATG
GATGCAGGTCGAGGCGATGCGGTGCTATCGGCCCTCGTTGTCGAACGCCACGTCG
TCCTCTCGGCGACCGTCAATCCTGGAAGGAAATGACCAAGCGTGCACCCAGCATC
CAGCATCCAACATCCAGCACCCAGCACCTAGCACCCATGATCCAATGGCCGGCATG
CAGATCAGCGTGGGGAGAGCACCATCTAGCAGCCCCTGGCAGGCCCAGCATTGTG
CTCGGATGTGCGATAGGCGGTGGGTGCGATAAGGTGGATTAGTGGAGGATGGAGG
ACTGAGTGGCGGGCAAGGGAAGGGGGCGGGAAGATGACTCACCTTGGGCAGTGG

GTGGGGGATGCGGTATAGGGGATGTATGTGGATGCTGGCGGAGGATAGAAGCTGT
AAGCTGAGCTGGTGCCAACGGTGACGCTGTGAGAGTGGTCTCGGCGACCCTGAC
GGCGACTCGGCGTTACCCCGTGTGGCACACCGGACGCGCGTCGAGTGACGTTGC
TGCTATTGTTGTTACCATCATCCTCTCCTCCGACAACCATACACTCAGTGCACACCAT
GCCACTCGAATCCTGCATGCTTATGTGAGTCCTCCCCTTCCCGTGCGCCGCTCTCC
AGCTCACATCCTCGCAGCGCGGCCGCTTAAAAACGGGTGGCTGCGCTGCTGCTGG
ATGTATGGGAAAGATGGAGAGATTGTAGGCTCGGCGGACTGATTTTCGAAACAGCT
AAGAAACGAGAACCACTACCGACAGTCCGGTTTTTCTTCCAGAAGCGGACATGACC
GCTGTTCGCGCGACGATGCCACTCCTTCATTCAGCAAGAATGAAGCAAGGAAGAAC
GATGTCATTCCGCACTCTTGGGAAGGAATCCACCATCAACAAAAGGTATGGAATAAA
ACAGATGCTGTGTTGCTAGGAGCTACCTTTTGTTTCTCATAAAAGATTGTCTATACATA
TATGGATAATACTAGGCATTGTCCGCTTCAGAGCCAAGAGAACAAACGGCTGTTTCT
CTACATGCCATGCATACTTGTATATATGGATAGCGCCCCTGCACCGCACCACGATCC
TCATCATTACATATCTTCTCTTCCAAGTATGCTGCATATATAGCTCTCGATGCGGTACG
CATCGTTGATTGAAACGCTTGGTGGAGTGCTTGATGGCCCGAAATGCCACGTGTAC
AACGCCACAAGCCCGAAAGGTAGGGCCTCGTGTAAAACGCCGCGATTCGCCGGCC
CTCCGTGGTCCCTGTTCGTTCCGTCTctctccctggcgccctcgtcctcccccactcccaacatctctttctt
ccttcatcttccaactccctcaaatccatactcccctctctcaccactcagccaaaccaaaaatgtcccgactcgacaagtc
caaggtcatcaactccgccctcgagctcctcaacgaggtcggtatcgagggtctcaccacccgaaagctcgcccagaag
ctcggtgtcgagcagcccaccctctactggcacgtcaagaacaagcgagccctcctcgacgccctcgccatcgagatgc
tcgaccgacaccacacccacttctgtcccctcgagggtgagtcctggcaggacttcctccgaaacaacgccaagtccttc
cgatgtgccctcctctcccaccgagacggtgccaaggtccacctcggtacccgacccaccgagaagcagtacgagacc
ctcgagaaccagctcgccttcctctgtcagcagggtttctccctcgagaacgccctctacgccctctccgccgtcggtcactt
caccctcggttgtgtcctcgaggaccaggagcaccaggtcgccaaggaggagcgagagacccccaccaccgactcca
tgccccccctcctccgacaggccatcgagctcttcgaccaccagggtgccgagcccgccttcctcttcggtctcgagctcat
catctgtggtctcgagaagcagctcaagtgtgagtccggttcctccaccctcttcggtggtgccaacttcaaccagtccggta
acatcgccgactcctccctctccttcaccttcaccaactcctccaacggtcccaacctcatcaccacccagaccaactccc
aggccctctcccagcccatcgcctcctccaacgtccacgacaacttcatgaacaacaccgaccagaccgcctacaacg
ccttcggtatcaccaccggtatgttcaacaccaccaccatggacgacgtctacaactacctcttcgacgacgaggacacc
ccccccaaccccaagaaggagtaagctagagataatcaagttgataaatggttgtgattagctgtaacaatcattg

3. pLC2060

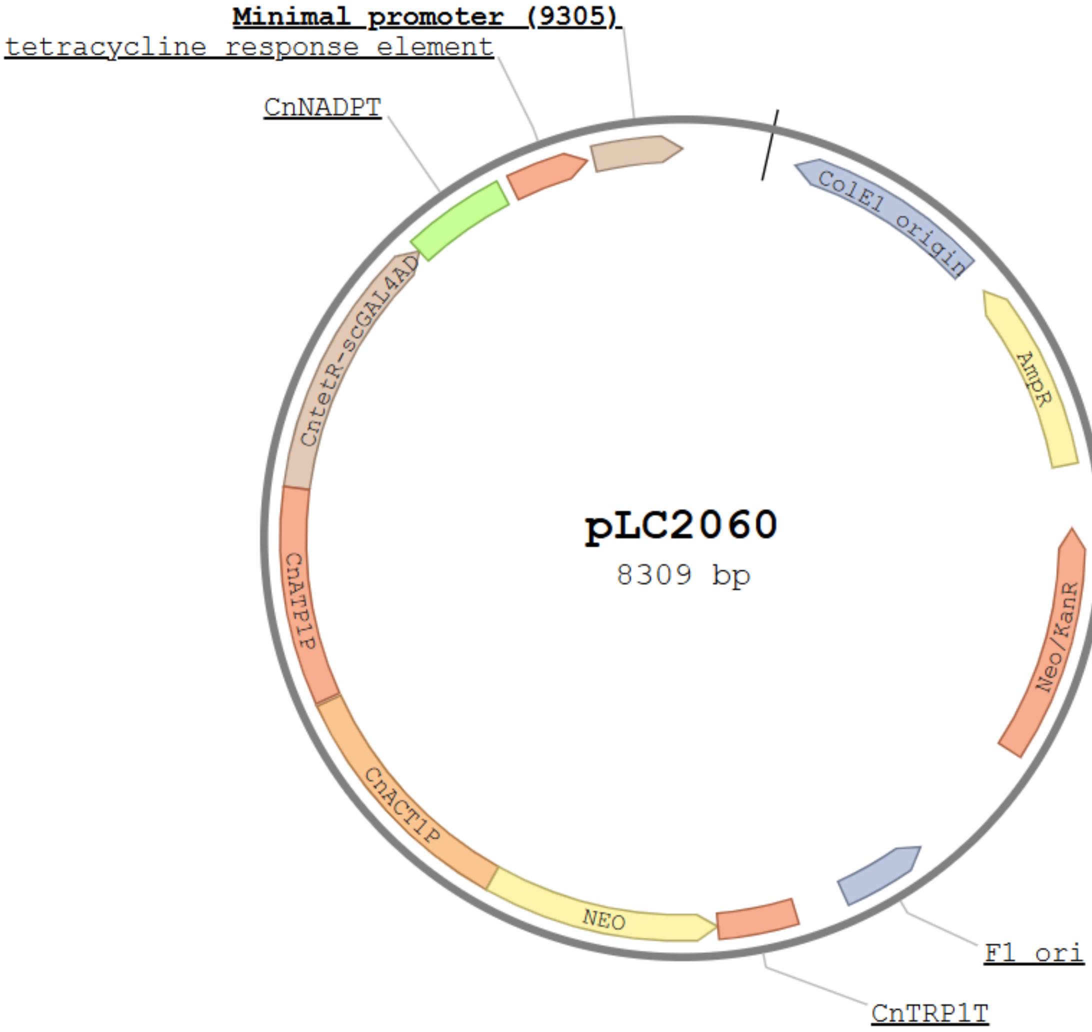

>pLC2060

catggtcatagctgtttcctgtgtgaaattgttatccgctcacaattccacacaacatacgagccggaagcataaagtgtaaa
gcctggggtgcctaatgagtgagctaactcacattaattgcgttgcgctcactgcccgctttccagtcgggaaacctgtcgtg
ccagctgcattaatgaatcggccaacgcgcggggagaggcggtttgcgtattgggcgctcttccgcttcctcgctcactgac
tcgctgcgctcggtcgttcggctgcggcgagcggtatcagctcactcaaaggcggtaatacggttatccacagaatcagg
ggataacgcaggaaagaacatgtgagcaaaaggccagcaaaaggccaggaaccgtaaaaaggccgcgttgctggc
gtttttccataggctccgcccccctgacgagcatcacaaaaatcgacgctcaagtcagaggtggcgaaacccgacagga
ctataaagataccaggcgtttccccctggaagctccctcgtgcgctctcctgttccgaccctgccgcttaccggatacctgtc
cgcctttctcccttcgggaagcgtggcgctttctcatagctcacgctgtaggtatctcagttcggtgtaggtcgttcgctccaag
ctgggctgtgtgcacgaaccccccgttcagcccgaccgctgcgccttatccggtaactatcgtcttgagtccaacccggtaa

gacacgacttatcgccactggcagcagccactggtaacaggattagcagagcgaggtatgtaggcggtgctacagagtt
cttgaagtggtggcctaactacggctacactagaagaacagtatttggtatctgcgctctgctgaagccagttaccttcggaa
aaagagttggtagctcttgatccggcaaacaaaccaccgctggtagcggtggtttttttgtttgcaagcagcagattacgcg
cagaaaaaaaggatctcaagaagatcctttgatcttttctacggggtctgacgctcagtggaacgaaaactcacgttaagg
gattttggtcatgagattatcaaaaaggatcttcacctagatccttttaaattaaaaatgaagttttaaatcaatctaaagtatat
atgagtaaacttggtctgacagttaccaatgcttaatcagtgaggcacctatctcGgcgatctgtctatttcgttcatccatagtt
gcctgactccccgtcgtgtagataactacgatacgggagggcttaccatctggccccagtgctgcaatgataccgcgaga
cccacgctcaccggctccagatttatcagcaataaaccagccagccggaagggccgagcgcagaagtggtcctgcaa
ctttatccgcctccatccagtctattaattgttgccgggaagctagagtaagtagttcgccagttaatagtttgcgcaacgttgtt
gccattgctacaggcatcgtggtgtcacgctcgtcgtttggtatggcttcattcagctccggttcccaacgatcaaggcgagtt
acatgatcccccatgttgtgcaaaaaagcggttagctccttcggtcctccgatcgttgtcagaagtaagttggccgcagtgtt
atcactcatggttatggcagcactgcataattctcttactgtcatgccatccgtaagatgcttttctgtgactggtgagtactcaa
ccaagtcattctgagaatagtgtatgcggcgaccgagttgctcttgcccggcgtcaatacgggataataccgcgccacata
gcagaactttaaaagtgctcatcattggaaaacgttcttcggggcgaaaactctcaaggatcttaccgctgttgagatccag
ttcgatgtaacccactcgtgcacccaactgatcttcagcatcttttactttcaccagcgtttctgggtgagcaaaaacaggaa
ggcaaaatgccgcaaaaaagggaataagggcgacacggaaatgttgaatactcatactcttcctttttcaattcagaaga
actcgtcaagaaggcgatagaaggcgatgcgctgcgaatcgggagcggcgataccgtaaagcacgaggaagcggtc
agcccattcgccgccaagctcttcagcaatatcacgggtagccaacgctatgtcctgatagcggtccgccacacccagcc
ggccacagtcgatgaatccagaaaagcggccattttccaccatgatattcggcaagcaggcatcgccatgggtcacgac
gagatcctcgccgtcgggcatgcgcgccttgagcctgAcgaacagttcggctggcgcgagcccctgatgctcttcgtcca
gatcatcctgatcgacaagaccggcttccatccgagtacgtgctcgctcgatgcgatgtttcgcttggtggtcgaatgggca
ggtagccggatcaagcgtatgcagccgccgcattgcatcagccatgatggatactttctcggcaggagcaaggtgggatg
acaggagatcctgccccggcacttcgcccaatagcagccagtcccttcccgcttcagtgacaacgtcgagcacagctgc
gcaaggaacgcccgtcgtggccagccacgatagccgcgctgcctcgtcctgcagttcattcagggcaccggacaggtc
ggtcttgacaaaaagaaccgggcgcccctgcgctgacagccggaacacggcggcatcagagcagccgattgtctgttg
tgcccagtcatagccgaatagcctctccacccaagcggccggagaacctgcgtgcaatccatcttgttcaatcatgcgaa
acgatcctcatcctgtctcttgatcagatcttgatcccctgcgccatcagatccttggcggcaagaaagccatccagtttacttt
gcagggcttcccaaccttaccagagggcgccccagctggcaattccggttcgcttgctgtccataaaaccgcccagtctag
ctatcgccatgtaagcccactgcaagctacctgctttctctttgcgcttgcgttttcccttgtccagatagcccagtagctgacat
tcatccggggtcagcaccgtttctgcggactggctttctacgtgttccgcttcctttagcagcccttgcgccctgaatttgttaaa
attcgcgttaaatttttgttaaatcagctcattttttaaccaataggccgaaatcggcaaaatcccttataaatcaaaagaatag
accgagatagggttgagtgttgttccagtttggaacaagagtccactattaaagaacgtggactccaacgtcaaagggcg
aaaaaccgtctatcagggcgatggcccactacgtgaaccatcaccctaatcaagttttttggggtcgaggtgccgtaaagc
actaaatcggaaccctaaagggagcccccgatttagagcttgacggggaaagccggcgaacgtggcgagaaaggaa
gggaagaaagcgaaaggagcgggcgctagggcgctggcaagtgtagcggtcacgctgcgcgtaaccaccacaccc
gccgcgcttaatgcgccgctacagggcgcgtccattcgccattcaggctgcgcaactgttgggaagggcgatcggtgcgg
gcctcttcgctattacgccagctggcgaaagggggatgtgctgcaaggcgattaagttgggtaacgccagggttttcccagt
cacgacgttgtaaaacgacggccagtGGTTTATCTGTATTAACACGGAAGAGATGTAGAAACTA
GCTTCCTGGTTTCAGAGACAAGGAGCCATGAAGATCCTGAGGATTCAACGGCGTTG
CTGAGGGTGGGAAGGATGGTATTGTTGTCTGTAGATAGATTAATGGGCATATAATGT

CAAAAAAGATGATTGACACGATGTCTGTCTCGAATTCGCAGTGTGCTAAAATTGTAT
CACTTCTCTAATATGTATATATACACCCTCTAAGGAAAATTAACCCCTTACCGCCTTCA
CTCAGAAGAACTCGTCAAGAAGGCGATAGAAGGCGATGCGCTGCGAATCGGGAGC
GGCGATACCGTAAAGCACGAGGAAGCGGTCAGCCCATTCGCCGCCAAGCTCTTCA
GCAATATCACGGGTAGCCAACGCTATGTCCTGATAGCGGTCCGCCACACCCAGCCG
GCCACAGTCGATGAATCCAGAAAAGCGGCCATTTTCCACCATGATATTCGGCAAGC
AGGCATCGCCATGGGTCACGACGAGATCCTCGCCGTCGGGCATGCGCGCCTTGAG
CCTGGCGAACAGTTCGGCTGGCGCGAGCCCCTGATGCTCTTCGTCCAGATCATCC
TGATCGACAAGACCGGCTTCCATCCGAGTACGTGCTCGCTCGATGCGATGTTTCGC
TTGGTGGTCGAATGGGCAGGTAGCCGGATCAAGCGTATGCAGCCGCCGCATTGCA
TCAGCCATGATGGATACTTTCTCGGCAGGAGCAAGGTGGGATGACAGGAGATCCTG
CCCCGGCACTTCGCCCAATAGCAGCCAGTCCCTTCCCGCTTCAGTGACAACGTCG
AGCACAGCTGCGCAAGGAACGCCCGTCGTGGCCAGCCACGATAGCCGCGCTGCC
TCGTCCTGCAGTTCATTCAGGGCACCGGACAGGTCGATCTTGACAAAAAGAACCG
GGCGCCCCTGCGCTGACAGCCGGAACACGGCGGCATCAGAGCAGCCGATTGTCT
GTTGTGCCCAGTCATAGCCGAATAGCCTCTCCACCCAAGCGGCCGGAGAACCTGC
GTGCAATCCATCTTGTTCAATCATAGACATGTTGGGCGAGTTTACTAATGGAAAAAG
ACAATTAGCAAAGAGGCTGCATATTAGGAGACGCGGTGAAGATTGGGGAGGACAAT
AGGGTCCGTGCACCAAAAAGTCCTGACGGCAGCTCTGTTCAACGCACATCCATCTC
TTCCAGCTATGCGTGTGGCACCAGCACGGATGGATTGTTGCACCAAAAGGACAAG
GCGAGATGGATGCAGGTCGAGGCGATGCGGTGCTATCGGCCCTCGTTGTCGAACG
CCACGTCGTCCTCTCGGCGACCGTCAATCCTGGAAGGAAATGACCAAGCGTGCAC
CCAGCATCCAGCATCCAACATCCAGCACCCAGCACCTAGCACCCATGATCCAATGG
CCGGCATGCAGATCAGCGTGGGGAGAGCACCATCTAGCAGCCCCTGGCAGGCCC
AGCATTGTGCTCGGATGTGCGATAGGCGGTGGGTGCGATAAGGTGGATTAGTGGA
GGATGGAGGACTGAGTGGCGGGCAAGGGAAGGGGGCGGGAAGATGACTCACCTT
GGGCAGTGGGTGGGGGATGCGGTATAGGGGATGTATGTGGATGCTGGCGGAGGAT
AGAAGCTGTAAGCTGAGCTGGTGCCAACGGTGACGCTGTGAGAGTGGTCTCGGC
GACCCTGACGGCGACTCGGCGTTACCCCGTGTGGCACACCGGACGCGCGTCGAG
TGACGTTGCTGCTATTGTTGTTACCATCATCCTCTCCTCCGACAACCATACACTCAG
TGCACACCATGCCACTCGAATCCTGCATGCTTATGTGAGTCCTCCCCTTCCCGTGC
GCCGCTCTCCAGCTCACATCCTCGCAGCGCGGCCGCTTAAAAACGGGTGGCTGCG
CTGCTGCTGGATGTATGGGAAAGATGGAGAGATTGTAGGCTCGGCGGACTGATTTT
CGAAACAGCTAAGAAACGAGAACCACTACCGACAGTCCGGTTTTTCTTCCAGAAGC
GGACATGACCGCTGTTCGCGCGACGATGCCACTCCTTCATTCAGCAAGAATGAAGC
AAGGAAGAACGATGTCATTCCGCACTCTTGGGAAGGAATCCACCATCAACAAAAGG
TATGGAATAAAACAGATGCTGTGTTGCTAGGAGCTACCTTTTGTTTCTCATAAAAGAT
TGTCTATACATATATGGATAATACTAGGCATTGTCCGCTTCAGAGCCAAGAGAACAAA
CGGCTGTTTCTCTACATGCCATGCATACTTGTATATATGGATAGCGCCCCTGCACCG
CACCACGATCCTCATCATTACATATCTTCTCTTCCAAGTATGCTGCATATATAGCTCTC

GATGCGGTACGCATCGTTGATTGAAACGCTTGGTGGAGTGCTTGATGGCCCGAAAT
GCCACGTGTACAACGCCACAAGCCCGAAAGGTAGGGCCTCGTGTAAAACGCCGCG
ATTCGCCGGCCCTCCGTGGTCCCTGTTCGTTCCGTCTctctccctggcgccctcgtcctcccca
ctcccaacatctctttcttccttcatcttccaactccctcaaatccatactcccctctctcaccactcagccaaaccaaaaatgt
cccgactcgacaagtccaaggtcatcaactccgccctcgagctcctcaacgaggtcggtatcgagggtctcaccacccg
aaagctcgcccagaagctcggtgtcgagcagcccaccctctactggcacgtcaagaacaagcgagccctcctcgacgc
cctcgccatcgagatgctcgaccgacaccacacccacttctgtcccctcgagggtgagtcctggcaggacttcctccgaa
acaacgccaagtccttccgatgtgccctcctctcccaccgagacggtgccaaggtccacctcggtacccgacccaccga
gaagcagtacgagaccctcgagaaccagctcgccttcctctgtcagcagggtttctccctcgagaacgccctctacgccct
ctccgccgtcggtcacttcaccctcggttgtgtcctcgaggaccaggagcaccaggtcgccaaggaggagcgagagac
ccccaccaccgactccatgccccccctcctccgacaggccatcgagctcttcgaccaccagggtgccgagcccgccttc
ctcttcggtctcgagctcatcatctgtggtctcgagaagcagctcaagtgtgagtccggttcctccaccctcttcggtggtgcc
aacttcaaccagtccggtaacatcgccgactcctccctctccttcaccttcaccaactcctccaacggtcccaacctcatcac
cacccagaccaactcccaggccctctcccagcccatcgcctcctccaacgtccacgacaacttcatgaacaacaccgac
cagaccgcctacaacgccttcggtatcaccaccggtatgttcaacaccaccaccatggacgacgtctacaactacctcttc
gacgacgaggacacccccccaaccccaagaaggagtaagctagagataatcaagttgataaatggttgtgattagct
gtaacaatcattgaaaaaaaaaaaagaaaagaaggaacaggagaagaagggaagaagccggtatacagttatttgtt
cataaagaattttatcgcccgtatgcagtttatttgttcatcaagaatttcatCTTTTTATCTGTCACAGTAGCGA
GGTCTTCTTTTTTGAATTTTACCAGTATCGGCTCTTGATCAACCCTTGCCAGCTTCTA
GCCACATAATCGACAAAACCCATAGCGTCACCCACATTCTGCTCAGTGTTCTCTACC
GATTTCACCAGTTGCTCATAACGTCCCAGGTTCGTGCGAAATCGATgtttggttcagcacctt
gtcgagtttaccactccctatcagtgatagagaaaagtgaaagtcgagtttaccactccctatcagtgatagagaaaagtg
aaagtcgagtttaccactccctatcagtgatagagaaaagtgaaagtcgagtttaccactccctatcagtgatagagaaaa
gtgaaagtcgagtttaccactccctatcagtgatagagaaaagtgaaagtcgagtttaccactccctatcagtgatagaga
aaagtgaaagtcgagtttaccactccctatcagtgatagagaaaagtgaaagtcgagcACTAGTAGTCGGTTAT
GATGACTGTCCCAGAGAACTATGTCACTCATCATTGGTCTTGAAAGTTTCAGCCGGC
CATTCGGTTCGACGAGGCGGAGAGAAGTCACGCTCATAAGGGAAAACTAGCTACTT
CAGCAGCTTTAGCATTCGATTGACGACAGCAGccgtatttaatccatcccgtttccatcttccttccgcac
aactcaaaaaacaaatctctctcttctcatactccaaacccaccaccagtataatgtgagtatcgccaccgtcccccgaca
cacgcacccacgctcacgcctcacagcaac

4. pLC2061

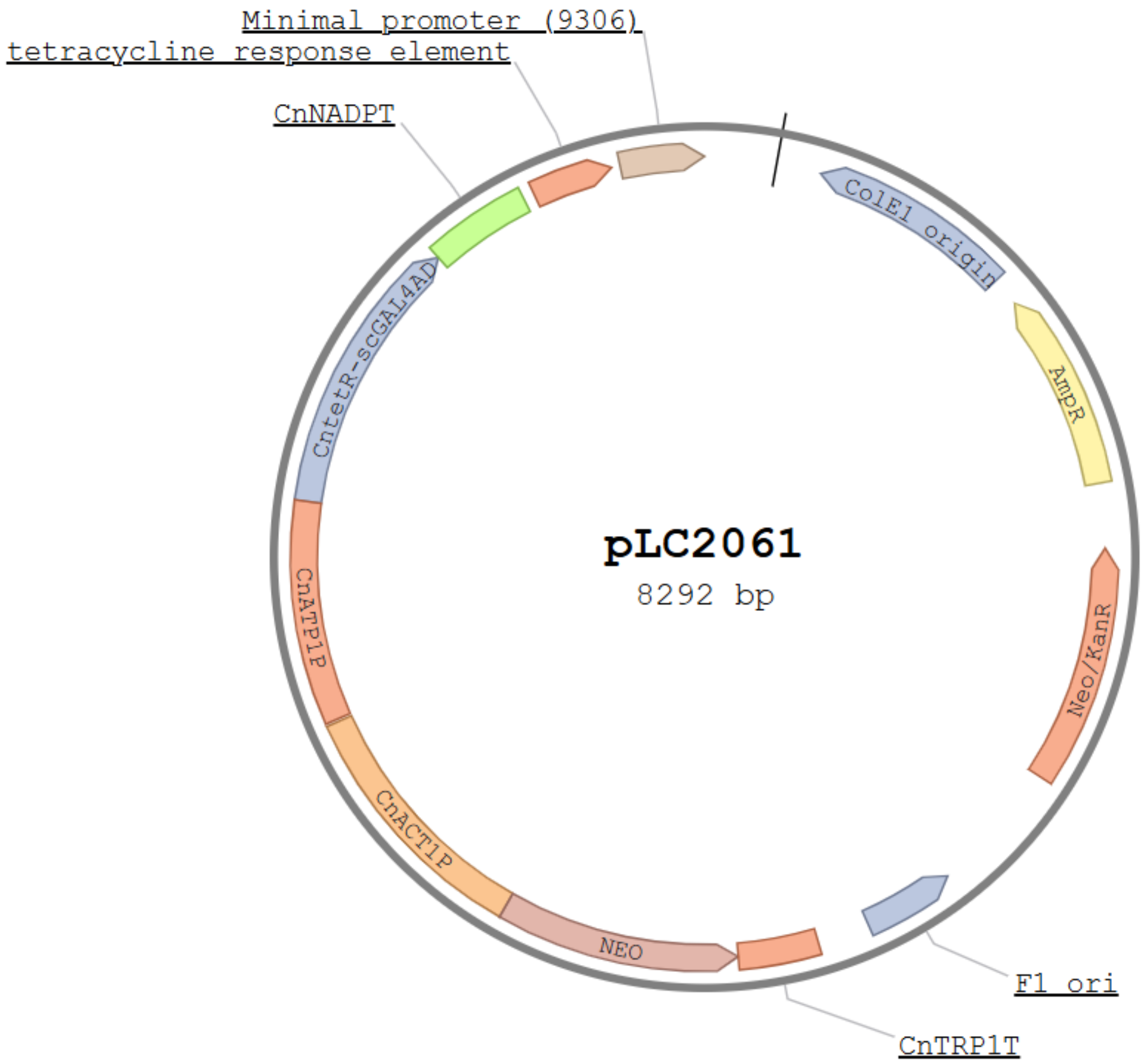

>pLC2061

catggtcatagctgtttcctgtgtgaaattgttatccgctcacaattccacacaacatacgagccggaagcataaagtgtaaa
gcctggggtgcctaatgagtgagctaactcacattaattgcgttgcgctcactgcccgctttccagtcgggaaacctgtcgtg
ccagctgcattaatgaatcggccaacgcgcggggagaggcggtttgcgtattgggcgctcttccgcttcctcgctcactgac
tcgctgcgctcggtcgttcggctgcggcgagcggtatcagctcactcaaaggcggtaatacggttatccacagaatcagg
ggataacgcaggaaagaacatgtgagcaaaaggccagcaaaaggccaggaaccgtaaaaaggccgcgttgctggc
gtttttccataggctccgcccccctgacgagcatcacaaaaatcgacgctcaagtcagaggtggcgaaacccgacagga
ctataaagataccaggcgtttccccctggaagctccctcgtgcgctctcctgttccgaccctgccgcttaccggatacctgtc
cgcctttctcccttcgggaagcgtggcgctttctcatagctcacgctgtaggtatctcagttcggtgtaggtcgttcgctccaag
ctgggctgtgtgcacgaaccccccgttcagcccgaccgctgcgccttatccggtaactatcgtcttgagtccaacccggtaa

gacacgacttatcgccactggcagcagccactggtaacaggattagcagagcgaggtatgtaggcggtgctacagagtt
cttgaagtggtggcctaactacggctacactagaagaacagtatttggtatctgcgctctgctgaagccagttaccttcggaa
aaagagttggtagctcttgatccggcaaacaaaccaccgctggtagcggtggtttttttgtttgcaagcagcagattacgcg
cagaaaaaaaggatctcaagaagatcctttgatcttttctacggggtctgacgctcagtggaacgaaaactcacgttaagg
gattttggtcatgagattatcaaaaaggatcttcacctagatccttttaaattaaaaatgaagttttaaatcaatctaaagtatat
atgagtaaacttggtctgacagttaccaatgcttaatcagtgaggcacctatctcGgcgatctgtctatttcgttcatccatagtt
gcctgactccccgtcgtgtagataactacgatacgggagggcttaccatctggccccagtgctgcaatgataccgcgaga
cccacgctcaccggctccagatttatcagcaataaaccagccagccggaagggccgagcgcagaagtggtcctgcaa
ctttatccgcctccatccagtctattaattgttgccgggaagctagagtaagtagttcgccagttaatagtttgcgcaacgttgtt
gccattgctacaggcatcgtggtgtcacgctcgtcgtttggtatggcttcattcagctccggttcccaacgatcaaggcgagtt
acatgatcccccatgttgtgcaaaaaagcggttagctccttcggtcctccgatcgttgtcagaagtaagttggccgcagtgtt
atcactcatggttatggcagcactgcataattctcttactgtcatgccatccgtaagatgcttttctgtgactggtgagtactcaa
ccaagtcattctgagaatagtgtatgcggcgaccgagttgctcttgcccggcgtcaatacgggataataccgcgccacata
gcagaactttaaaagtgctcatcattggaaaacgttcttcggggcgaaaactctcaaggatcttaccgctgttgagatccag
ttcgatgtaacccactcgtgcacccaactgatcttcagcatcttttactttcaccagcgtttctgggtgagcaaaaacaggaa
ggcaaaatgccgcaaaaaagggaataagggcgacacggaaatgttgaatactcatactcttcctttttcaattcagaaga
actcgtcaagaaggcgatagaaggcgatgcgctgcgaatcgggagcggcgataccgtaaagcacgaggaagcggtc
agcccattcgccgccaagctcttcagcaatatcacgggtagccaacgctatgtcctgatagcggtccgccacacccagcc
ggccacagtcgatgaatccagaaaagcggccattttccaccatgatattcggcaagcaggcatcgccatgggtcacgac
gagatcctcgccgtcgggcatgcgcgccttgagcctgAcgaacagttcggctggcgcgagcccctgatgctcttcgtcca
gatcatcctgatcgacaagaccggcttccatccgagtacgtgctcgctcgatgcgatgtttcgcttggtggtcgaatgggca
ggtagccggatcaagcgtatgcagccgccgcattgcatcagccatgatggatactttctcggcaggagcaaggtgggatg
acaggagatcctgccccggcacttcgcccaatagcagccagtcccttcccgcttcagtgacaacgtcgagcacagctgc
gcaaggaacgcccgtcgtggccagccacgatagccgcgctgcctcgtcctgcagttcattcagggcaccggacaggtc
ggtcttgacaaaaagaaccgggcgcccctgcgctgacagccggaacacggcggcatcagagcagccgattgtctgttg
tgcccagtcatagccgaatagcctctccacccaagcggccggagaacctgcgtgcaatccatcttgttcaatcatgcgaa
acgatcctcatcctgtctcttgatcagatcttgatcccctgcgccatcagatccttggcggcaagaaagccatccagtttactt
gcagggcttcccaaccttaccagagggcgccccagctggcaattccggttcgcttgctgtccataaaaccgcccagtctag
ctatcgccatgtaagcccactgcaagctacctgctttctctttgcgcttgcgttttcccttgtccagatagcccagtagctgacat
tcatccggggtcagcaccgtttctgcggactggctttctacgtgttccgcttcctttagcagcccttgcgccctgaatttgttaaa
attcgcgttaaatttttgttaaatcagctcattttttaaccaataggccgaaatcggcaaaatcccttataaatcaaaagaatag
accgagatagggttgagtgttgttccagtttggaacaagagtccactattaaagaacgtggactccaacgtcaaagggcg
aaaaaccgtctatcagggcgatggcccactacgtgaaccatcaccctaatcaagttttttggggtcgaggtgccgtaaagc
actaaatcggaaccctaaagggagcccccgatttagagcttgacggggaaagccggcgaacgtggcgagaaaggaa
gggaagaaagcgaaaggagcgggcgctagggcgctggcaagtgtagcggtcacgctgcgcgtaaccaccacaccc
gccgcgcttaatgcgccgctacagggcgcgtccattcgccattcaggctgcgcaactgttgggaagggcgatcggtgcgg
gcctcttcgctattacgccagctggcgaaagggggatgtgctgcaaggcgattaagttgggtaacgccagggttttcccagt
cacgacgttgtaaaacgacggccagtGGTTTATCTGTATTAACACGGAAGAGATGTAGAAACTA
GCTTCCTGGTTTCAGAGACAAGGAGCCATGAAGATCCTGAGGATTCAACGGCGTTG
CTGAGGGTGGGAAGGATGGTATTGTTGTCTGTAGATAGATTAATGGGCATATAATGT

CAAAAAAGATGATTGACACGATGTCTGTCTCGAATTCGCAGTGTGCTAAAATTGTAT
CACTTCTCTAATATGTATATATACACCCTCTAAGGAAAATTAACCCCTTACCGCCTTCA
CTCAGAAGAACTCGTCAAGAAGGCGATAGAAGGCGATGCGCTGCGAATCGGGAGC
GGCGATACCGTAAAGCACGAGGAAGCGGTCAGCCCATTCGCCGCCAAGCTCTTCA
GCAATATCACGGGTAGCCAACGCTATGTCCTGATAGCGGTCCGCCACACCCAGCCG
GCCACAGTCGATGAATCCAGAAAAGCGGCCATTTTCCACCATGATATTCGGCAAGC
AGGCATCGCCATGGGTCACGACGAGATCCTCGCCGTCGGGCATGCGCGCCTTGAG
CCTGGCGAACAGTTCGGCTGGCGCGAGCCCCTGATGCTCTTCGTCCAGATCATCC
TGATCGACAAGACCGGCTTCCATCCGAGTACGTGCTCGCTCGATGCGATGTTTCGC
TTGGTGGTCGAATGGGCAGGTAGCCGGATCAAGCGTATGCAGCCGCCGCATTGCA
TCAGCCATGATGGATACTTTCTCGGCAGGAGCAAGGTGGGATGACAGGAGATCCTG
CCCCGGCACTTCGCCCAATAGCAGCCAGTCCCTTCCCGCTTCAGTGACAACGTCG
AGCACAGCTGCGCAAGGAACGCCCGTCGTGGCCAGCCACGATAGCCGCGCTGCC
TCGTCCTGCAGTTCATTCAGGGCACCGGACAGGTCGATCTTGACAAAAAGAACCG
GGCGCCCCTGCGCTGACAGCCGGAACACGGCGGCATCAGAGCAGCCGATTGTCT
GTTGTGCCCAGTCATAGCCGAATAGCCTCTCCACCCAAGCGGCCGGAGAACCTGC
GTGCAATCCATCTTGTTCAATCATAGACATGTTGGGCGAGTTTACTAATGGAAAAAG
ACAATTAGCAAAGAGGCTGCATATTAGGAGACGCGGTGAAGATTGGGGAGGACAAT
AGGGTCCGTGCACCAAAAAGTCCTGACGGCAGCTCTGTTCAACGCACATCCATCTC
TTCCAGCTATGCGTGTGGCACCAGCACGGATGGATTGTTGCACCAAAAGGACAAG
GCGAGATGGATGCAGGTCGAGGCGATGCGGTGCTATCGGCCCTCGTTGTCGAACG
CCACGTCGTCCTCTCGGCGACCGTCAATCCTGGAAGGAAATGACCAAGCGTGCAC
CCAGCATCCAGCATCCAACATCCAGCACCCAGCACCTAGCACCCATGATCCAATGG
CCGGCATGCAGATCAGCGTGGGGAGAGCACCATCTAGCAGCCCCTGGCAGGCCC
AGCATTGTGCTCGGATGTGCGATAGGCGGTGGGTGCGATAAGGTGGATTAGTGGA
GGATGGAGGACTGAGTGGCGGGCAAGGGAAGGGGGCGGGAAGATGACTCACCTT
GGGCAGTGGGTGGGGGATGCGGTATAGGGGATGTATGTGGATGCTGGCGGAGGAT
AGAAGCTGTAAGCTGAGCTGGTGCCAACGGTGACGCTGTGAGAGTGGTCTCGGC
GACCCTGACGGCGACTCGGCGTTACCCCGTGTGGCACACCGGACGCGCGTCGAG
TGACGTTGCTGCTATTGTTGTTACCATCATCCTCTCCTCCGACAACCATACACTCAG
TGCACACCATGCCACTCGAATCCTGCATGCTTATGTGAGTCCTCCCCTTCCCGTGC
GCCGCTCTCCAGCTCACATCCTCGCAGCGCGGCCGCTTAAAAACGGGTGGCTGCG
CTGCTGCTGGATGTATGGGAAAGATGGAGAGATTGTAGGCTCGGCGGACTGATTTT
CGAAACAGCTAAGAAACGAGAACCACTACCGACAGTCCGGTTTTTCTTCCAGAAGC
GGACATGACCGCTGTTCGCGCGACGATGCCACTCCTTCATTCAGCAAGAATGAAGC
AAGGAAGAACGATGTCATTCCGCACTCTTGGGAAGGAATCCACCATCAACAAAAGG
TATGGAATAAAACAGATGCTGTGTTGCTAGGAGCTACCTTTTGTTTCTCATAAAAGAT
TGTCTATACATATATGGATAATACTAGGCATTGTCCGCTTCAGAGCCAAGAGAACAAA
CGGCTGTTTCTCTACATGCCATGCATACTTGTATATATGGATAGCGCCCCTGCACCG
CACCACGATCCTCATCATTACATATCTTCTCTTCCAAGTATGCTGCATATATAGCTCTC

GATGCGGTACGCATCGTTGATTGAAACGCTTGGTGGAGTGCTTGATGGCCCGAAAT
GCCACGTGTACAACGCCACAAGCCCGAAAGGTAGGGCCTCGTGTAAAACGCCGCG
ATTCGCCGGCCCTCCGTGGTCCCTGTTCGTTCCGTCTctctccctggcgccctcgtcctcccca
ctcccaacatctctttcttccttcatcttccaactccctcaaatccatactcccctctctcaccactcagccaaaccaaaaatgt
cccgactcgacaagtccaaggtcatcaactccgccctcgagctcctcaacgaggtcggtatcgagggtctcaccacccg
aaagctcgcccagaagctcggtgtcgagcagcccaccctctactggcacgtcaagaacaagcgagccctcctcgacgc
cctcgccatcgagatgctcgaccgacaccacacccacttctgtcccctcgagggtgagtcctggcaggacttcctccgaa
acaacgccaagtccttccgatgtgccctcctctcccaccgagacggtgccaaggtccacctcggtacccgacccaccga
gaagcagtacgagaccctcgagaaccagctcgccttcctctgtcagcagggtttctccctcgagaacgccctctacgccct
ctccgccgtcggtcacttcaccctcggttgtgtcctcgaggaccaggagcaccaggtcgccaaggaggagcgagagac
ccccaccaccgactccatgccccccctcctccgacaggccatcgagctcttcgaccaccagggtgccgagcccgccttc
ctcttcggtctcgagctcatcatctgtggtctcgagaagcagctcaagtgtgagtccggttcctccaccctcttcggtggtgcc
aacttcaaccagtccggtaacatcgccgactcctccctctccttcaccttcaccaactcctccaacggtcccaacctcatcac
cacccagaccaactcccaggccctctcccagcccatcgcctcctccaacgtccacgacaacttcatgaacaacaccgac
cagaccgcctacaacgccttcggtatcaccaccggtatgttcaacaccaccaccatggacgacgtctacaactacctcttc
gacgacgaggacaCccccccccaaccccaagaaggagtaagctagagataatcaagttgataaatggttgtgattagc
tgtaacaatcattgaaaaaaaaaaaagaaaagaaggaacaggagaagaagggaagaagccggtatacagttatttgt
tcataaagaattttatcgcccgtatgcagtttatttgttcatcaagaatttcatCTTTTTATCTGTCACAGTAGCGA
GGTCTTCTTTTTTGAATTTTACCAGTATCGGCTCTTGATCAACCCTcGCCAGCTTCTA
GCCACATAATCGACAAAACCCATAGCGTCACCCACATTCTGCTCAGTGTTCTCTACC
GATTTCACCAGTTGCTCATAACGTCCCAGGTTCGTGCGAAATCGATgtttggttcagcacctt
gtcgagtttaccactccctatcagtgatagagaaaagtgaaagtcgagtttaccactccctatcagtgatagagaaaagtg
aaagtcgagtttaccactccctatcagtgatagagaaaagtgaaagtcgagtttaccactccctatcagtgatagagaaaa
gtgaaagtcgagtttaccactccctatcagtgatagagaaaagtgaaagtcgagtttaccactccctatcagtgatagaga
aaagtgaaagtcgagtttaccactccctatcagtgatagagaaaagtgaaagtcgagcACTAGTTGGAAGATG
GAGATATGGTACAAGAATGGACAGATGGAAACAAGAAATGGGCATCGCTCCTTTAG
GGTGGATACGTGAGTTTTTGTCAGAGATGAGTGGAAACCCAAAATGACTGTCATCG
TAGTCCCGATCGGTAGccgtatttaatccatcccgtttccatcttccttccgcacaactcaaaaaacaatctctctc
ttctcatactccaaacccaccaccagtataatgtgagtatcgccaccgtcccccgacacacgcacccacgctcacgcctc
acagcaac

5. Clustal alignment of codon optimized *CntetR-ScGAL4AD* sequence in adapted *tetO* plasmids for *Cryptococcus neoformans* with the adapted *tetR-ScGAL4AD* sequence for *Candida albicans*.

```
CLUSTAL O(1.2.4) multiple sequence alignment


CatetR-ScGAL4AD(pLC1031)            atgtctagattagataaaagtaaagtgattaacAgcgcattagagttgcttaatgAggtc 60
CntetR-ScGAL4AD(pLC1774-1775)       atgtcccgactcgacaagtccaaggtcatcaactccgccctcgagctcctcaacgaggtc 60
                                    *****  ** * ** **    ** ** ** ***  ***  * *** * ** ** ******

CatetR-ScGAL4AD(pLC1031)            ggaatcgaaggtttaacaacccgtaaactcgcccagaagctaggtgtagagcagcctaca 120
CntetR-ScGAL4AD(pLC1774-1775)       ggtatcgagggtctcaccacccgaaagctcgcccagaagctcggtgtcgagcagcccacc 120
                                    ** ***** *** * ** ***** ** ************** ***** ******** **

CatetR-ScGAL4AD(pLC1031)            ttgtattggcatgtaaaaaataagcgggctttgctcgacgccttaGcCattgagatgtta 180
CntetR-ScGAL4AD(pLC1774-1775)       ctctactggcacgtcaagaacaagcgagccctcctcgacgccctcgccatcgagatgctc 180
                                     * ** ***** ** ** ** ***** **  * ********* * ***** ****** *

CatetR-ScGAL4AD(pLC1031)            gataggcaccatactcacttttgccctttagaaggggaaagctggcaagattttttacgt 240
CntetR-ScGAL4AD(pLC1774-1775)       gaccgacaccacacccacttctgtcccctcgagggtgagtcctggcaggacttcctccga 240
                                    **  * ***** ** ***** ** **  * ** ** **   ****** ** **  * **

CatetR-ScGAL4AD(pLC1031)            aataacgctaaaagttttagatgtgctttactaagtcatcgcgatggagcaaaagtacat 300
CntetR-ScGAL4AD(pLC1774-1775)       aacaacgccaagtccttccgatgtgccctcctctcccaccgagacggtgccaaggtccac 300
                                    ** ***** **    ** *******  * **    ** ** ** ** ** ** ** **

CatetR-ScGAL4AD(pLC1031)            ttaggtacacggcctacagaaaaacagtatgaaactctcgaaaatcaattagccttttta 360
CntetR-ScGAL4AD(pLC1774-1775)       ctcggtacccgacccaccgagaagcagtacgagaccctcgagaaccagctcgccttcctc 360
                                     * ***** ** ** ** ** ** ***** ** ** ***** ** **  * *****  *

CatetR-ScGAL4AD(pLC1031)            tgccaacaaggtttttcactagagaatgcattatatgcactcagcgctgtggggcatttt 420
CntetR-ScGAL4AD(pLC1774-1775)       tgtcagcagggtttctccctcgagaacgccctctacgccctctccgccgtcggtcacttc 420
                                    ** ** ** ***** ** ***** **  * ** ** ***  *** ** ** ** **

CatetR-ScGAL4AD(pLC1031)            actttaggttgcgtattggaagaTcaagagcatcaagtcgctaaagaagaaagggaaaca 480
CntetR-ScGAL4AD(pLC1774-1775)       accctcggttgtgtcctcgaggaccaggagcaccaggtcgccaaggaggagcgagagacc 480
                                    **  * ***** **  * ** ** ** ***** ** ***** ** ** **  * ** **

CatetR-ScGAL4AD(pLC1031)            cctactactgatagtatgccgccattattacgacaagctatcgaattatttgatCACcaa 540
CntetR-ScGAL4AD(pLC1774-1775)       cccaccaccgactccatgccccccctcctccgacaggccatcgagctcttcgaccaccag 540
                                    ** ** ** **    ***** **  *  * ***** ** *****  * ** ** *****

CatetR-ScGAL4AD(pLC1031)            ggtgcagagccagccttcttattcggccttgaattgatcatatgcggattagaaaaacaa 600
CntetR-ScGAL4AD(pLC1774-1775)       ggtgccgagcccgccttcctcttcggtctcgagctcatcatctgtggtctcgagaagcag 600
                                    ***** ***** ****** * ***** ** **  * ***** ** **  * ** ** **

CatetR-ScGAL4AD(pLC1031)            cttaaatgtgaaagtgggtcctcgactttgtttggtggcgccaattttaatcaaagtggg 660
CntetR-ScGAL4AD(pLC1774-1775)       ctcaagtgtgagtccggttcctccaccctcttcggtggtgccaacttcaaccagtccggt 660
                                    ** ** *****    ** ***** **  * ** ***** ***** ** ** **    **

CatetR-ScGAL4AD(pLC1031)            aatattgctgatagctcattgtccttcactttcactaacagtagcaacggtccgaacctc 720
CntetR-ScGAL4AD(pLC1774-1775)       aacatcgccgactcctccctctccttcaccttcaccaactcctccaacggtcccaacctc 720
                                    ** ** ** **   ***  * ******** ***** ***     ********* ******

CatetR-ScGAL4AD(pLC1031)            ataacaactcaaacaaattctcaagcgctttcacaaccaattgcctcctctaacgttcat 780
CntetR-ScGAL4AD(pLC1774-1775)       atcaccacccagaccaactcccaggccctctcccagcccatcgcctcctccaacgtccac 780
                                    ** ** ** ** ** ** ** ** ** ** ** ** ** ** ******** ***** **

CatetR-ScGAL4AD(pLC1031)            gataacttcatgaataatacggaccaaactgcgtataacgcgtttggaatcactacaggg 840
CntetR-ScGAL4AD(pLC1774-1775)       gacaacttcatgaacaacaccgaccagaccgcctacaacgccttcggtatcaccaccggt 840
                                    ** *********** ** ** ***** ** ** ** ***** ** ** ***** ** **

CatetR-ScGAL4AD(pLC1031)            atgtttaataccactacaatggatgatgtatataactatctattcgatgatgaagatacc 900
CntetR-ScGAL4AD(pLC1774-1775)       atgttcaacaccaccaccatggacgacgtctacaactacctcttcgacgacgaggacacc 900
                                    ***** ** ***** ** ***** ** ** ** ***** ** ***** ** ** ** ***

CatetR-ScGAL4AD(pLC1031)            ccaccaaacccaaaaaaagagtaa      924
CntetR-ScGAL4AD(pLC1774-1775)       ccccccaaccccaagaaggagtaa      924
                                    ** ** ***** ** ** ******
```
